# Structure and Spin-Glass Magnetism of the Fe_1.5_Ni_1.5_Ga_4_ Metallic Alloy

**DOI:** 10.1021/acs.inorgchem.5c05447

**Published:** 2026-02-20

**Authors:** Krishnendu Buxi, Rahul Pan, Zvonko Jagličić, Andreja Jelen, Jože Luzar, Peter Mihor, Stanislav Vrtnik, Primož Koželj, Julia Petrović, Maxim Avdeev, Partha Pratim Jana, Janez Dolinšek

**Affiliations:** † Department of Chemistry, 30133 Indian Institute of Technology, Kharagpur 721302, India; ‡ Institute of Mathematics, Physics and Mechanics & University of Ljubljana, Faculty of Civil and Geodetic Engineering, Jadranska 19, SI-1000 Ljubljana, Slovenia; § 61790 Jožef Stefan Institute, Jamova 39, SI-1000 Ljubljana, Slovenia; ∥ University of Ljubljana, Faculty of Mathematics and Physics, Jadranska 19, SI-1000 Ljubljana, Slovenia; ⊥ 5419 Australian Nuclear Science and Technology Organisation, New Illawarra Road, Lucas Heights, NSW 2234, Australia; # School of Chemistry, The University of Sydney, Sydney, NSW 2006, Australia

## Abstract

The Ga-rich region of the Fe–Ni–Ga ternary system was investigated, by exploring a line of compositions Fe_*x*_Ni_3–*x*_Ga_4_, with 0.5 ≤ *x* ≤ 2.5. The single-phase cubic material was found only at the composition Fe_1.5_Ni_1.5_Ga_4_ and its immediate vicinity, representing a new phase in the Fe–Ni–Ga diagram. The homogeneity range of this phase was estimated by additionally exploring a set of compositions Fe_*x*_Ni_*y*_Ga_*z*_ around the central composition Fe_1.5_Ni_1.5_Ga_4_. The structural model was constructed based on the structure of the binary Ni_3_Ga_4_ parent phase, which crystallizes in the cubic *Ia*3̅*d* space group. We have considered that by substituting Fe for Ni, the *Ia*3̅*d* structure is preserved, with the Fe and Ni being statistically distributed at their 48*g* Wyckoff site. The possibility of a symmetry-reduced chiral structural model *I*4_1_32 driven by chemical ordering of Fe and Ni cannot be entirely ruled out on the basis of the crystallographic study. The magnetic study of the Fe_1.5_Ni_1.5_Ga_4_ phase has revealed that the material forms a spin glass phase below the spin freezing temperature *T*_f_ ≈ 9 K. Since the spin glass ordering of the Fe and Ni magnetic moments is compatible with their random distribution, the magnetic study supports the disordered cubic *Ia*3̅*d* model.

## Graphical abstract

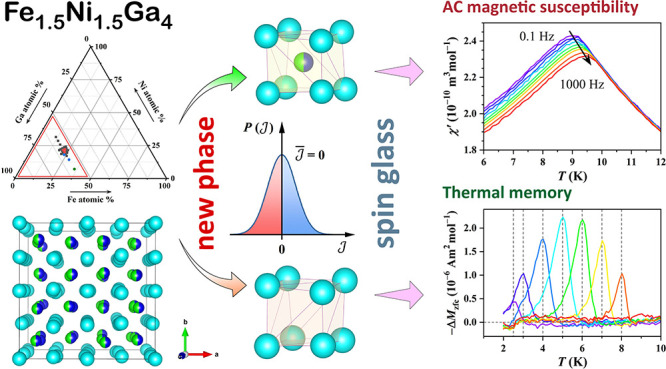

## 1 Introduction

Intermetallic compounds of transition metals with gallium (Ga) are well-known for their diverse structural variations and interesting physical/chemical properties. The group of gallides includes compounds with TiNiSi-type structure (ScNiGa, ScPtGa, ScAuGa), ZrBeSi-type NbRhGa, MgZn_2_-type NbCr_1.58_Ga_0.42_ and NbFe_1.51_Ga_0.49_,^1^ Ti_2–*x*_Ni_3_Ga_9_ ternary intermetallics with a distorted HoCoGa_5_-type structure,^2^ various gallium-based Heusler alloys of the type X_2_ZGa, with X and Z standing for 3*d* transition metals, including ferromagnetic Co_2_ZGa series,^3^ superconducting ReGa_5_,^4^ magnetic α-Fe_6_Ga_5_ and GaMn,^5,6^ magnetocaloric MnNiGa_2_ full-Heusler alloy^7^ and other magnetocaloric alloys in the Ni–Mn–Ga system^8–11^ and thermoelectric Fe_2_CoGa Heusler alloy.^12^ Bimetallic compounds of Ga with transition metals show superior catalytic properties in heterogeneous catalysis. The Pd_m_Ga_n_ series (PdGa, Pd_2_Ga, Pd_3_Ga_7_) are highly selective and stable catalyst materials for the selective hydrogenation of alkynes and the methanol steam reforming reaction,^13–16^ while noble metal-free intermetallic compounds in the Ni–Ga system (NiGa, Ni_2_Ga_3_, Ni_3_Ga, Ni_5_Ga_3_) were reported to reduce CO_2_ to methanol at ambient pressure.^17–19^

Recently, a novel chiral intermetallic compound Co_3_Ni_3_Ga_8_ was reported,^20^ obtained by substituting half of the Ni atoms by Co in the binary parent compound Ni_3_Ga_4_ (*Ia*3̅*d*, *cI*112).^21,22^ The substitution induces local distortions in the lattice, driven by chemical ordering of Ni and Co, which makes the Co_3_Ni_3_Ga_8_ compound to adopt a lower-symmetric chiral structure *I*4_1_32, as compared to the parent Ni_3_Ga_4_. The report^20^ also provides a theoretical prediction of antiferromagnetic spin ordering in both the binary Ni_3_Ga_4_ and its ternary derivative Co_3_Ni_3_Ga_8_. An interesting feature emerges in the electron density of states diagrams. As the number of electrons in the Co_3_Ni_3_Ga_8_ is reduced with respect to the binary Ni_3_Ga_4_, the *d*-band of the transition metal atoms shifts closer to the Fermi level.^20^ These results motivated us to investigate the Fe substitution for Ni in the Ni_3_Ga_4_ for two reasons: (1) Fe, due to its slightly larger atomic-size mismatch relative to Ni may induce larger local distortions than Co, potentially leading to interesting structural transformations and (2) Fe substitution further reduces the electron count of the system, which may shift the *d*-band of the transition metals closer to the Fermi level, opening the door to novel magnetic and transport properties.

According to the Fe–Ni–Ga ternary phase diagram,^23^ the Ga-poor region contains at least two intermetallic phases, the Heusler alloy Fe_2_NiGa (*Fm*3̅*m*, *cF*16)^24^ and the monoclinic FeNi_2_Ga (*C*2/*m*, *mS*40).^25^ These compounds were identified as ferromagnetic shape memory alloys.^26^ The Ga-rich part of the ternary system remains largely unexplored. In this course of investigation, the Ga-rich region of the Fe–Ni–Ga ternary system is explored by studying a line of compositions at a constant Ga content Fe_*x*_Ni_3–*x*_Ga_4_, with 0.5 ≤ *x* ≤ 2.5. Single-phase material was found only at the composition Fe_1.5_Ni_1.5_Ga_4_ and its immediate vicinity. The existence range of the Fe_1.5_Ni_1.5_Ga_4_ phase was estimated by additionally exploring a set of compositions Fe_*x*_Ni_*y*_Ga_*z*_, where the concentrations of all three elements were varied independently around the central composition Fe_1.5_Ni_1.5_Ga_4_. We are reporting on the crystal structure and magnetic properties of the cubic Fe_1.5_Ni_1.5_Ga_4_ phase, representing a new phase in the Fe–Ni–Ga ternary system. The structural model is constructed based on the structure of binary Ni_3_Ga_4_.^21,22^ Like the binary Ni_3_Ga_4_, the structure is considered to crystallize in the cubic *Ia*3̅*d* space group, where Fe substitutes Ni to form Fe_1.5_Ni_1.5_Ga_4_. Here it is noteworthy that, though the Fe_3_Ga_4_ in the Fe–Ga system^27–29^ has a similar composition to Ni_3_Ga_4_, it adopts a monoclinic structure (*C*2/*m*, *mS*42). A ferrimagnetic ordering in the low Ni-doped solid solution of the Fe_3_Ga_4_ has been reported.^30^ The possibility of a symmetry-reduced chiral structural model *I*4_1_32 of the Fe_1.5_Ni_1.5_Ga_4_ phase, driven by chemical ordering between Fe and Ni cannot be entirely ruled out on the basis of the crystallographic study, because of the weak X-ray scattering contrast between Fe and Ni and their similar neutron coherent scattering lengths. The magnetic study, on the other hand, supports the *Ia*3̅*d* structural model without chemical ordering of Fe and Ni, because the Fe_1.5_Ni_1.5_Ga_4_ phase behaves magnetically at low temperatures as a spin glass, compatible with statistical distribution of the Fe and Ni atomic magnetic moments in a single Wyckoff site.

## 2 Material Synthesis and Structural Characterization

### 2.1 Synthesis

Ten Fe_*x*_Ni_3–*x*_Ga_4_ compositions with nominal Fe contents *x* = 0.5, 0.8, 1.0, 1.2, 1.4, 1.5, 1.6, 1.8, 2.0, and 2.5, as well as 14 compositions Fe_*x*_Ni_*y*_Ga_*z*_ with 1.3 ≤ *x* ≤ 1.74, 1.26 ≤ *y* ≤ 1.7 and 3.8 ≤ *z* ≤ 4.2 were synthesized from pure constituent elements nickel powder (99.995%), gallium metal (99.9%) and iron granules (1–2 mm, 99.98%), all from Alfa Aesar, using high-temperature synthesis technique. Given that gallium metal remains liquid at room temperature (RT), handling requires additional precautions. Gallium was solidified over ice and promptly transferred into a one-end-sealed silica tube of diameter ∼ 8 mm. Nickel powder and iron granules were weighted and transferred. The tubes were connected to a vacuum sealing unit, and pure argon gas was purged into the evacuated tubes multiple times to remove the trace amount of air. Then the samples were sealed in vacuum (∼10^–6^ mbar) to avoid metal oxide formation at elevated temperatures. The sealed tubes were placed into a cylindrical alumina crucible covered with sand-sized quartz clasts to buffer against the temperature fluctuations during heat treatment. The alumina crucibles were placed into a muffle furnace, which was ramped up to 1223 K at a rate of 62 Kh^–1^ and held at that temperature for 17 h, followed by cooling to 723 K at a rate of 9 Kh^–1^. The samples were dwelled at that temperature for a prolonged time (∼8–9 days). Then the samples were cooled to 473 K at a rate of 4 Kh^–1^, and at this stage the furnace was turned off and allowed to cool to ambient temperature. The large-scale samples (4 g for neutron powder diffraction) were prepared in an ampule of a diameter ∼ 12 mm, using longer annealing time (∼9–10 days) at 723 K. The ingots obtained after the reactions were air-stable with a shiny metallic luster. The ingots were broken with an agate mortar and pestle into small crystallites, which were then used for further characterization.

### 2.2 Structure Solution and Refinement by Single-Crystal X-ray Diffraction

The single-crystal X-ray diffraction (SCXRD) data collection and processing are detailed in the Experimental section. The data collected for the Fe_1.5_Ni_1.5_Ga_4_ crystal were indexed based on *a* = 11.5192 Å cubic *I*-centered unit cell and the *hk*0 and *hhl* planes in the reciprocal space are shown in Figure S1 of the Supporting Information. The extinction conditions in the reciprocal space suggested the space group *Ia*3̅*d* (No. 230). The charge flipping algorithm^31^ in JANA2006 ^32^ yielded three independent crystallographic sites, 16*a*, 48*f*, and 48*g*, among which 16*a* and 48*f* were occupied by Ga, and 48*g* was occupied by Fe. The refinement converged to *R*_obs_ ∼ 0.048. An independent refinement of the Fe position resulted in the site occupancy factor (SOF) greater than unity; therefore, this site was examined for Ni occupancy and subsequent refinement resulted in SOF (Ni) less than one. Hence, the 48*g* site was modeled as statistically mixed between Fe and Ni, and the SOF was fixed according to the nominal composition, assuming complete occupancy at that site. The refinement at this stage resulted in *R*_obs_ ∼ 0.040. In the next step, the atomic sites were refined anisotropically, and the refinement applying isotropic extinction correction yielded *R*_obs_ ∼ 0.017 and goodness-of-fit (GOF) of 1.33. No significant electron density was found on the difference Fourier map. The X-ray crystallographic data on the structure solution and refinement are detailed in Table 1, while the atomic coordinates, site occupancies and isotropic displacement parameters are given in Table 2. The structural data are also available as a CIF file, deposited in the Cambridge Crystallographic Data Centre (CCDC) as the number CSD 2502085.

**Table 1:** Crystallographic Data Collection and Refinement for the Fe_1.5_Ni_1.5_Ga_4_ Single Crystal (CSD Deposition Number 2502085)

crystallographic data	
chemical formula	Fe_1.5_Ni_1.5_Ga_4_
chemical formula (at.%)	Fe_21.43_Ni_21.43_Ga_57.14_
EDS formula (at.%)	Fe_19.5_Ni_22.2_Ga_58.3_
crystal system	cubic
space group; *Z*	*Ia*3̅*d* (No. 230); 16
*a*/Å	11.5192(4)
*V*/Å^3^	1528.51(9)
ρ_calc_/g cm^–3^	7.8339
μ/mm^–1^	40.29
*F*(000)	3280
crystal color	silvery with metallic luster
data collection	four-circle diffractometer
diffractometer	Bruker Photon II
radiation; wavelength/Å	Mo Kα; 0.71073
monochromator	graphite
*T*/K	294(2)
θ_min_ – θ_max_/deg	4.33–36.17
reflns measured	18,460
index range	–19 ≤ *h* ≤ 19
	–19 ≤ *k* ≤ 19
	–19 ≤ *l* ≤ 19
data reduction/abs. correction	multiscan
crystal size/mm	0.09 × 0.07 × 0.02
unique reflns	312
*R*_int_	0.0551
structure solution/refinement	JANA2006 package program
structure solution	superflip
no. reflns used	4626
no. variables	14
observed reflns (*I* > 3σ(*I*))	259
*R*(*F* ^2^ > 3σ(*F* ^2^))	0.0171
*R*(*F*) (all data)	0.0231
*wR*(*F* ^2^) (all data)	0.0453
GOF (all)	1.33
Δρ_min_/Δρ_max_ (eÅ^–3^)	–0.75/0.51

**Table 2:** Atomic Coordinates, Site Occupancy Factors (SOF), and Equivalent Isotropic Displacement Parameters for the Fe_1.5_Ni_1.5_Ga_4_ Single Crystal

atom	Wyckoff	*x*	*y*	*z*	SOF	[Table-fn t2fn1] *U*_*eq*_ /Å^2^
Ga1	16*a*	0	0	0	1	0.00852(6)
Ga2	48*f*	1/4	0.01408(3)	0	1	0.00866(7)
Ni1/Fe1	48*g*	0.63462(2)	0.38462(2)	3/8	0.5/0.5	0.00816(7)

* *U*_eq_/Å^2^ is defined as one-third of the trace of the orthogonalized *U* ^*ij*^ tensor.

### 2.3 EDS and Elemental Mapping Analysis

Energy dispersive X-ray spectroscopy (EDS) compositional point analysis averaged over ten points yielded the composition (in at %, rounded to first integers) Fe_20_Ni_22_Ga_58_, with about ±0.5 at. % uncertainty for each element, in good agreement with the loading composition Fe_21.43_Ni_21.43_Ga_57.14_ of the Fe_1.5_Ni_1.5_Ga_4_ phase. EDS elemental maps of a larger piece of material show homogeneous distribution of the elements on a 100-μm scale (Figure S2).

### 2.4 Neutron Powder Diffraction Analysis

Due to the very weak X-ray scattering contrasts between Fe, Ni and Ga elements, an accurate distribution of the atoms cannot be determined based on the electron density only. Hence, a combination of X-ray and neutron powder diffraction (NPD) experiments was employed to overcome this problem. NPD allows accurate determination of the Ga site as the neutron coherent scattering length *b* of Ga (*b*_Ga_ = 7.288 fm) is distant enough from Fe (*b*_Fe_ = 9.45 fm) and Ni (*b*_Ni_ = 10.3 fm), whereas the site occupancy pattern of Fe and Ni cannot be reliably determined due to their too similar neutron coherent scattering lengths. The intensity of additional diffraction peaks due to complete Fe and Ni ordering and the splitting of the original Wyckoff site 48*g* in the *Ia*3̅*d* space group into 24*h* and 24*g* sites in the *I*4_1_32 would be on the scale of 0.8% of the strongest peak, which is below the detection level for the collected data. The Rietveld refinement of the NPD data at RT for the *Ia*3̅*d* model using JANA2006 software^32^ is shown in Figure 1a (the refinement parameters are given in the figure caption).

**Figure 1:**
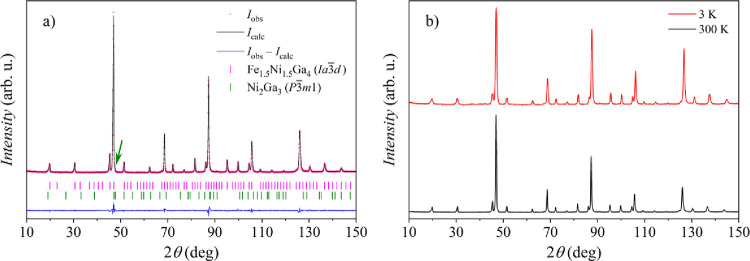
(a) Rietveld refinement of the Fe_1.5_Ni_1.5_Ga_4_ NPD data collected at 300 K (*R*_obs_ = 0.0244, *wR*_obs_ = 0.0297, *R*_p_ = 0.0484, *wR*_p_ = 0.0622, GOF = 1.49). *I*_obs_ is the observed intensity, *I*_calc_ the calculated intensity, and *I*_obs_ – *I*_calc_ is their difference. Since the large-scale synthesis of the Fe_1.5_Ni_1.5_Ga_4_ material for the NPD study has yielded a trace amount of Ni_2_Ga_3_ as an impurity phase (its most intense peak is marked by an arrow), the Rietveld refinement was performed by taking into account both Fe_1.5_Ni_1.5_Ga_4_ (*Ia*3̅*d*) and Ni_2_Ga_3_ (*P*3̅*m*1) phases and their weight fractions were determined to be about ∼99 and ∼1 wt %. Bragg positions of both phases are shown by tick marks. (b) A comparison of the NPD data collected at 3 and 300 K temperatures.

The analysis supports the cubic *Ia*3̅*d* model, but is still inconclusive with regard to the possible chemical ordering of Fe and Ni, which could lead to the symmetry-reduced chiral structural model *I*4_1_32. A comparison of the NPD data collected at 3 and 300 K is shown in Figure 1b. No new (magnetic) peaks or intensity enhancements at low 2*θ* angles could be detected at 3 K in addition to the nuclear peaks, indicating the absence of long-range magnetic ordering down to that temperature. The variation of the peak intensity at higher 2*θ* angles for the 3 K data compared to the 300 K data can be attributed to the Debye–Waller factor.^33^

### 2.5 Phase Analysis by Powder X-ray Diffraction

The phase width has been analyzed by powder X-ray diffraction (PXRD) study. The PXRD data refinements (Rietveld and Le Bail) were performed employing JANA2006 software.^32^ The Rietveld refinement diffractogram for the Fe_1.5_Ni_1.5_Ga_4_ is depicted in Figure 2, while the diffractograms of the refinements (Rietveld or Le Bail) for other synthesized samples in the Fe_*x*_Ni_3–*x*_Ga_4_ series are given in Figure S3 of the Supporting Information (the refinement parameters are detailed in Table S1). Single-phase material with the *Ia*3̅*d* structure (and generic phase name Fe_1.5_Ni_1.5_Ga_4_) was found only for the compositions *x* = 1.5 and 1.6 (corresponding to Fe_21.43_Ni_21.43_Ga_57.14_ and Fe_22.86_Ni_20.00_Ga_57.14_, in at.%), while at other compositions, two to four phases were present (Table S1). For the compositions 0.8 ≤ *x* ≤ 1.8, the major phase was the cubic *Ia*3̅*d*, whereas for *x* = 2.0 and 2.5, the major phase was monoclinic *C*2/*m.*

**Figure 2:**
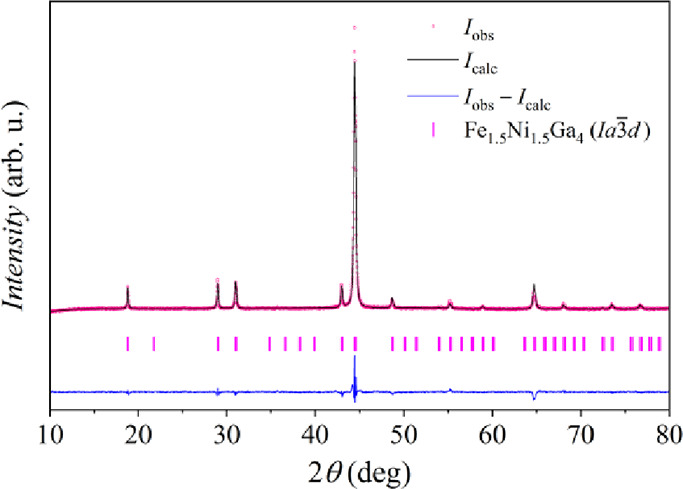
Rietveld refinement diffractogram of the PXRD pattern of Fe_1.5_Ni_1.5_Ga_4_ (*R*_obs_ = 0.0889, *wR*_obs_ = 0.1023, *R*_p_ = 0.0189, *wR*_p_ = 0.0292, GOF = 1.88). Bragg positions of the Fe_1.5_Ni_1.5_Ga_4_ (*Ia*3̅*d*) phase are shown by tick marks.

The refined PXRD diffractograms of the 14 Fe_*x*_Ni_*y*_Ga_*z*_ samples are shown in Figure S4, whereas the refinement parameters are given in Table S2 of the Supporting Information. For this series, single-phase cubic *Ia*3̅*d* material was found only at the composition Fe_22.06_Ni_22.06_Ga_55.88_ (i.e., Fe_1.5_Ni_1.5_Ga_3.8_), while for other compositions, two to four phases were present (Table S2), with the *Ia*3̅*d* being the major phase for all compositions except one (the Fe_23.54_Ni_18.01_Ga_58.45_ or Fe_1.65_Ni_1.26_Ga_4.09_). For that particular composition, the major phase was the monoclinic *C*2/*m*, while the *Ia*3̅*d* phase was not detected.

The phase analysis discerns that the cubic *Ia*3̅*d* phase exists as a single phase only in a narrow homogeneity range around the Fe_1.5_Ni_1.5_Ga_4_ composition, shown in the Fe–Ni–Ga ternary phase triangle of Figure 3. Other investigated compositions are multiphase (from two up to four phases), where the cubic *Ia*3̅*d* is the major phase for most compositions. The exceptions are the compositions Fe_28.57_Ni_14.29_Ga_57.14_ (Fe_2_NiGa_4_) and Fe_23.54_Ni_18.01_Ga_58.45_ (Fe_1.65_Ni_1.26_Ga_4.09_), where the major phase is the monoclinic *C*2/*m*, whereas the composition Fe_35.71_Ni_7.14_Ga_57.14_ (Fe_2.5_Ni_0.5_Ga_4_) is a single-phase *C*2/*m.*

**Figure 3:**
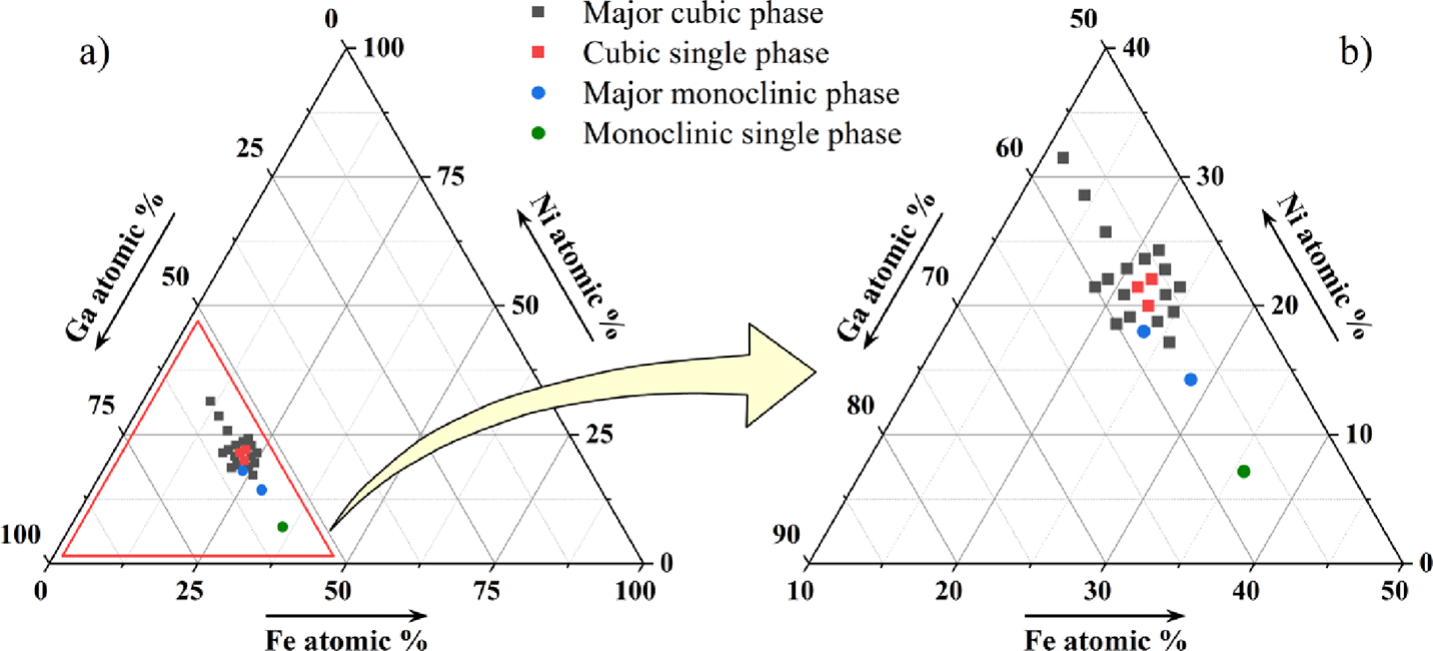
(a) Fe–Ni–Ga ternary phase triangle. (b) Ga-rich portion of the phase triangle magnified, with various symbols denoting the investigated compositions (the legend is written in the figure). Red squares denote the single-phase cubic *Ia*3̅*d* compositions.

### 2.6 Fe_1.5_Ni_1.5_Ga_4_ Structure Description

The parent compound Ni_3_Ga_4_ (*Ia*3̅*d*, *a* = 11.411 Å) (Figure 4a) can be described as a 3D-network of face-sharing Ga_8_ cubes extending along the crystallographic axes, where 3/4 of the cubic voids are occupied by Ni (Figure 4b) (Ni@Ga8) and the remaining 1/4 of them remain vacant (*V*@Ga8) (Figure 4c).^21,22^ Therefore, Ni_3_Ga_4_ can be considered as a *lacunar* CsCl-type phase. The volumes of the occupied and vacant Ga_8_ cubes are equal (*V*_Ni @ Ga8_ = *V*_V @ Ga8_= 23.61 Å^3^). Consequently, each Ni atom is surrounded by four other Ni atoms within the bonding distances (∼2.85 Å) (Figure 4d).

**Figure 4:**
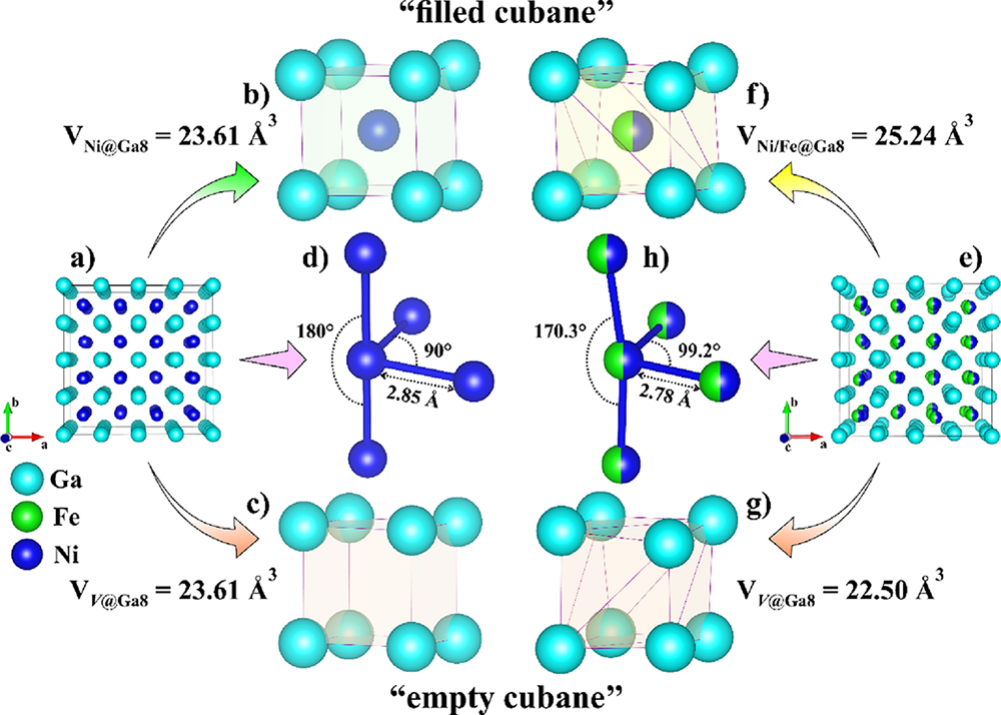
Structural description of (a–d) Ni_3_Ga_4_ and (e–h) Fe_1.5_Ni_1.5_Ga_4_ (for details, see the text).

This arrangement can be correlated with the electronic stability of this compound, which adheres to the 18-*n* rule to achieve a closed shell electronic configuration, where *n* represents the number of electron pairs that the atom shares with its neighboring transition metal atoms,^34^ and in this case *n* = 4. Upon replacing half of the Ni atoms with Fe, the unit cell parameter of the Fe_1.5_Ni_1.5_Ga_4_ increases, and certain distortions are introduced into the system without breaking any symmetry element (Figure 4e). As a result, the “filled cubane” expands in volume, *V*_Ni/Fe @ Ga8_= 25.24 Å^3^ (Figure 4f), whereas the “empty cubane” contracts, *V*_V @ Ga8_ = 22.50 Å^3^ (Figure 4g), driven by a reduction in the (Ni/Fe)–(Ni/Fe) bond lengths (∼2.78 Å) (Figure 4h) relative to the Ni–Ni bonds in the parent phase. The 18-*n* rule also breaks down, as achieving the closed-cell electronic configuration for the Fe_1.5_Ni_1.5_Ga_4_ would require each Ni/Fe atom to share 5 electron pairs with the neighboring atoms, i.e., to have five Ni/Fe neighbors within the bonding distances, which is clearly not the case.

## 3 Spin Glass Magnetism of the Fe_1.5_Ni_1.5_Ga_4_ Compound

Magnetic properties of an Fe_1.5_Ni_1.5_Ga_4_ polycrystalline sample were determined in the temperature interval between 300 and 1.8 K in magnetic fields up to 7 T, including low magnetic fields of the order 0.1–1 mT. The Fe_1.5_Ni_1.5_Ga_4_ compound contains two magnetic elements, Fe and Ni, both ferromagnetic (FM) as the elemental metals and both occupying the same Wyckoff site in the crystal structure. Since the compound is an electrical conductor, the interspin interaction is the Ruderman–Kittel–Kasuya–Yosida (RKKY) indirect exchange, mediated by the conduction electrons. This long-range interaction oscillates in sign between positive and negative with the interspin distance on the nanometric scale, and consequently there exist both FM and antiferromagnetic (AFM) interactions in the spin system. If the Fe and Ni moments would be randomly distributed over their crystallographic site, magnetic frustration of the spin system without long-range magnetic ordering can be expected, while in the case that Fe and Ni would be chemically ordered, the magnetic state could be long-range ordered. All magnetic results presented in the following that are given in units per mol were calculated per mole of Fe_0.214_Ni_0.214_Ga_0.572_ “average atoms” with the molar mass of 64.4 g mol^–1^.

### 3.1 Temperature-Dependent dc and ac Magnetic Susceptibility

Direct-current (dc) magnetic susceptibility χ = *M*/*H* was determined for both zero-field-cooled (zfc) and field-cooled (fc) protocols. The χ_zfc_ and χ_fc_ susceptibilities in the magnetic field μ_0_ *H* = 0.1 T in the entire investigated temperature range 300–1.8 K are shown in Figure 5, whereas the susceptibilities in various magnetic fields between 0.1 mT and 1 T on an expanded temperature scale below 20 K are shown in the inset.

**Figure 5:**
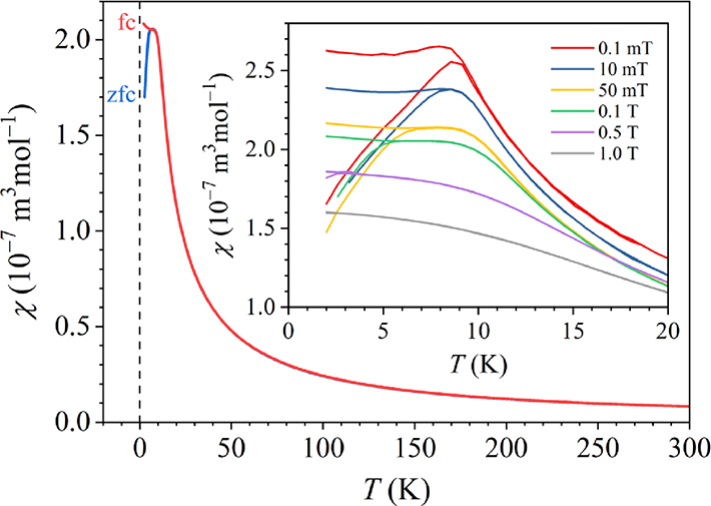
Zfc and fc dc magnetic susceptibilities, χ_zfc_ and χ_fc_, in the temperature interval 300–1.8 K in the magnetic field μ_0_ *H* = 0.1 T. The inset shows the susceptibilities in various magnetic fields 0.1 mT–1 T on an expanded temperature scale below 20 K.

Upon cooling from RT, the susceptibilities exhibit a 1/*T* Curie-type paramagnetic growth down to about 9 K, with no difference between χ_zfc_ and χ_fc_. At 9 K, χ_zfc_ exhibits a cusp and decreases toward zero upon further cooling, while χ_fc_ remains constant. These features are characteristic of a spin-freezing transition in a spin glass (SG).^35^ The spin-freezing temperature *T*_f_ is conveniently defined as the temperature of the χ_zfc_ maximum. Below *T*_f_, spin fluctuation times become macroscopically long and can no longer drive the spin system to thermal equilibrium on the available experimental time scale, so that the system becomes nonergodic. Broken ergodicity is the origin of the χ_zfc_ – χ_fc_ splitting.

The strength of the exchange interaction responsible for the formation of the SG magnetic structure was estimated by analyzing the shift of the χ_fc_ – χ_zfc_ bifurcation temperature (that equals *T*_f_ in the lowest field) to lower temperatures with increasing magnetic field. A continuous shift of the bifurcation temperature with the field is observed (inset in Figure 5), where in the field of 0.5 T, a tiny χ_fc_ – χ_zfc_ splitting is still observed at the lowest temperature of 1.8 K, while there is no more difference between χ_zfc_ and χ_fc_ in a 1 T field. This signals that the Zeeman (single-spin) interaction dominates over the exchange (two-spin) interaction and polarizes the spins along the magnetic field direction, whereas the frustrated magnetic structure that has been formed in zero field is destroyed.

The susceptibility in the paramagnetic regime (at *T* > *T*_f_ ≈ 9 K) was analyzed by the Curie–Weiss law, χ = χ_0_ + *C*/(*T* – θ), pertinent to an ensemble of localized paramagnetic moments. Here *C* is the Curie–Weiss constant, θ is the Curie–Weiss temperature and χ_0_ is the temperature-independent term (a sum of the negative Larmor diamagnetic susceptibility χ_Larmor_ of the atomic cores, the negative Landau diamagnetic susceptibility χ_Landau_ due to the conduction-electron circulation in an external magnetic field and the positive Pauli spin susceptibility χ_Pauli_ of the conduction electrons, where in the independent-electron model, all three contributions are of the same order of magnitude). The susceptibility in a 0.1 T field is shown in Figure 6 in a (χ – χ_0_)^−1^ vs *T* plot. The Curie–Weiss fit performed in the high-temperature regime for *T* > 100 K is shown by a solid line and the fit parameters are *C* = 2.5 × 10^–6^ m^3^ Kmol^–1^, θ = – 2.9 K and χ_0_= −1.3 × 10^–10^ m^3^ mol^–1^.

**Figure 6:**
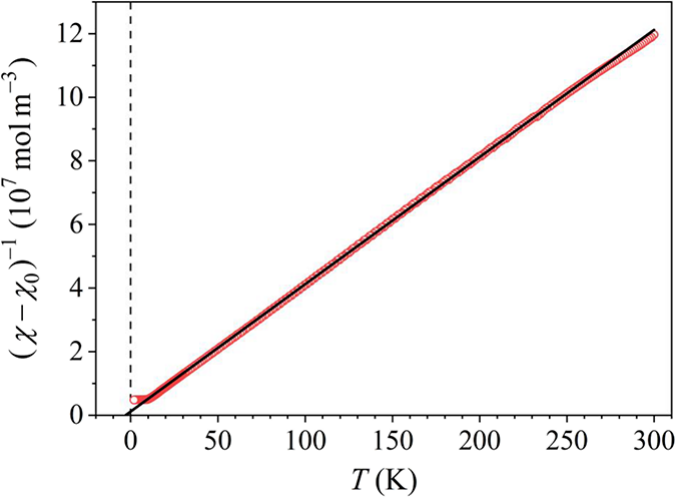
Magnetic susceptibility χ_fc_ in the field μ_0_ *H* = 0.1 T presented in a (χ – χ_0_)^−1^ vs *T* plot. The solid line is the Curie–Weiss paramagnetic fit at temperatures *T* > 100 K (the values of the fit parameters are given in the text).

The Curie–Weiss constant allows determination of the mean effective paramagnetic moment μ̅_eff_ = *p̅*_eff_μ_B_ (where μ_B_ is the Bohr magneton and *p̅*_eff_ is the mean effective Bohr magneton number) from the formula ${\overset{-}{p}}_{\mathrm{eff}}=(797.7\sqrt{\mathrm{mol}/m^{3}K})\sqrt{C}$ ,^36^ yielding μ̅_eff_ = 1.93 μ_B_ per one Fe_0.5_Ni_0.5_ “average” magnetic atom. This value is significantly reduced relative to the experimental paramagnetic free-ion values of the localized ions Fe^3+^, Fe^2+^, Ni^3+^, and Ni^2+^ that amount to 5.9, 5.4, 4.8, and 3.2 μ_B_, respectively. Such reduction is typical for electrically conducting paramagnets and originates from partial screening of the localized moments by the spins of conduction electrons. The fit-determined χ_0_ value was compared to the theoretical Larmor core susceptibility that was calculated from literature tables^37^ for different ionization states of the Fe and Ni elements to be in the interval χ_Larmor_ = [−1.3, −0.9] × 10^–10^ m^3^ mol^–1^, confirming that χ_0_ is of correct order of magnitude. The fact that χ_0_ is negative indicates dominance of the two diamagnetic contributions (Larmor and Landau) over the Pauli paramagnetic spin susceptibility.

The Curie–Weiss temperature θ is an indication of the dominant type of interspin interactions, being either FM (θ > 0) or AFM (θ < 0). In 3*d* magnetic alloys containing randomness, random local magnetic anisotropy at the atomic scale is generally insufficient to pin the local magnetization direction, so that the alloys are exchange-dominated spin systems. Randomness introduces a continuous distribution P(J) of the exchange coupling constants J , which extends on both J>0 (FM coupling) and J<0 (AFM coupling) sides of the J axis.^38^ A SG-type P(J) is, by definition, characterized by a zero average exchange interaction, J®=0 and consequently θ value close to zero. The negative θ= −2.9 K value of the Curie–Weiss temperature suggests dominant AFM-type interactions, but is small enough to support the SG-type magnetic state below *T*_f_ ≈ 9 K, where the FM and AFM interactions are both present in similar proportions.

Alternating-current (ac) susceptibility was measured in a sinusoidal magnetic field of amplitude μ_0_ *H* = 0.2 mT at logarithmically spaced frequencies in the interval ν = 0.1 Hz–1 kHz. The temperature-dependent real part of the susceptibility χ′ in the region of the spin-freezing transition is shown in Figure 7. χ′ exhibits a peak at *T*_f_, which shifts to higher temperatures with increasing frequency and the peak height slightly decreases. Such behavior is characteristic for a transition to a nonergodic spin state and the temperature of the χ′ peak can be conveniently defined as the frequency-dependent spin-freezing temperature *T*_f_(ν). At the lowest frequency, *T*_f_(0.1 Hz)= 9.0 K, while at the highest frequency, *T*_f_(1 kHz) = 9.5 K. The *T*_f_(ν)/*T*_f_(0.1 Hz) relation is shown in the inset of Figure 7, yielding the fractional shift of *T*_f_(ν) per decade of frequency Γ = Δ*T*_f_/*T*_f_Δ­(log ν) = 1.0 × 10^–2^. This value is typical for SGs, where Γ values in the range 10^–2^–10^–3^ are common.^39^

**Figure 7:**
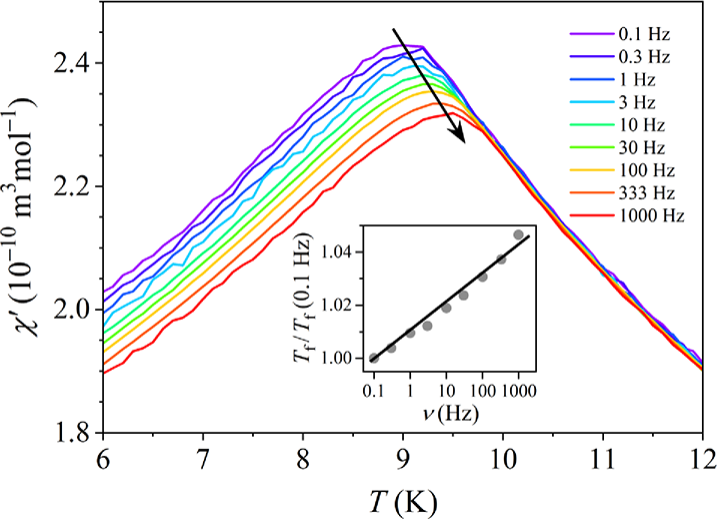
Real part χ′ of the ac magnetic susceptibility at frequencies in the interval ν = 0.1 Hz–1 kHz in the temperature range of the spin-freezing transition. The inset shows the *T*_f_(ν)/*T*_f_(0.1 Hz) relation on the logarithmic frequency scale.

### 3.2 Magnetization Versus Magnetic Field Curves

The magnetization versus the magnetic field, *M*(*H*), curves were determined at temperatures between 300 and 1.8 K for the field sweep μ_0_ *H*= ± 7 T and the curves at selected temperatures are shown in Figure 8a. The magnetization *M* is presented in units of μ_B_ per one Fe_0.214_Ni_0.214_Ga_0.572_ “average atom” of the Fe_1.5_Ni_1.5_Ga_4_ phase. Hysteresis appears below *T*_f_, as shown on an expanded scale in Figure 8b. The coercive field *H*_c_ increases monotonously upon cooling (inset in Figure 8b), reaching the value μ_0_ *H*_c_ = 39 mT at the lowest temperature of 1.8 K. The hysteresis curves close up in a field of about 1.5 T, as typical for an AFM-type hysteresis (the FM-type hysteresis curves typically close up in about 10-times lower field). In the large-field limit, the *M*(*H*) curves saturate to an inclined linear line.

**Figure 8:**
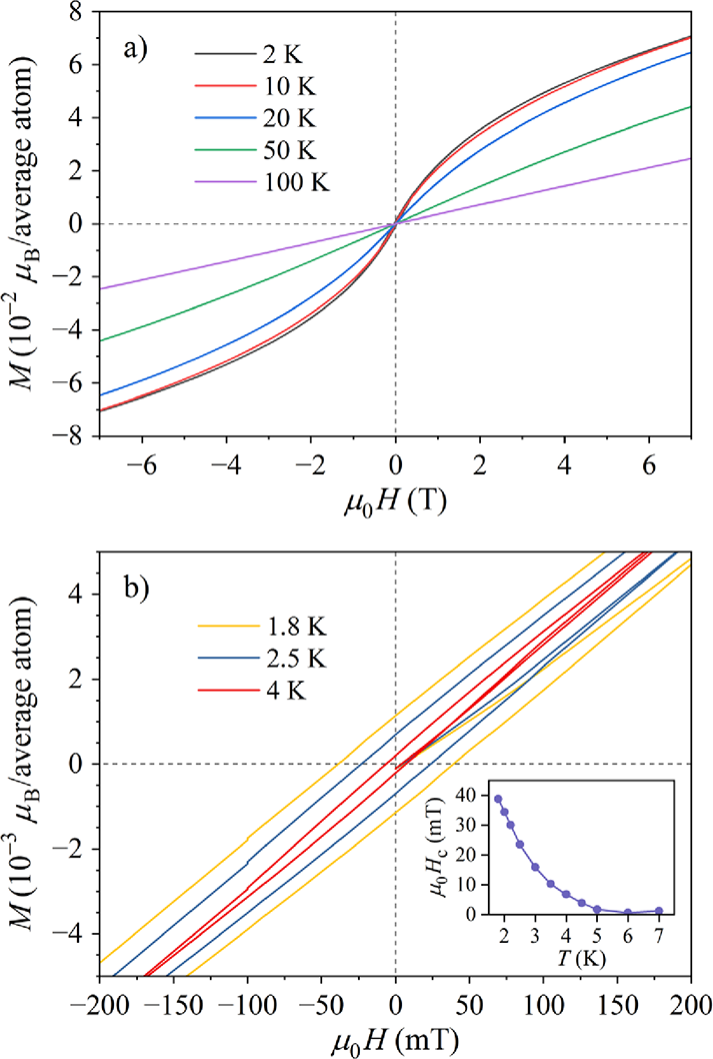
(a) *M*(*H*) curves at selected temperatures below and above the spin-freezing temperature *T*_f_ ≈ 9 K. (b) The *M*(*H*) curves below *T*_f_ expanded around the origin *H* = 0 to show the hysteretic region. The temperature-dependent coercive field *H*_c_ is presented in the inset.

The *M*(*H*) curve at *T* = 5 K, which is inside the SG phase, but the hysteresis is still small, was theoretically analyzed by assuming a SG-type continuous and symmetric distribution of the exchange coupling constants P(J) , shown schematically in the inset in Figure 9.

**Figure 9:**
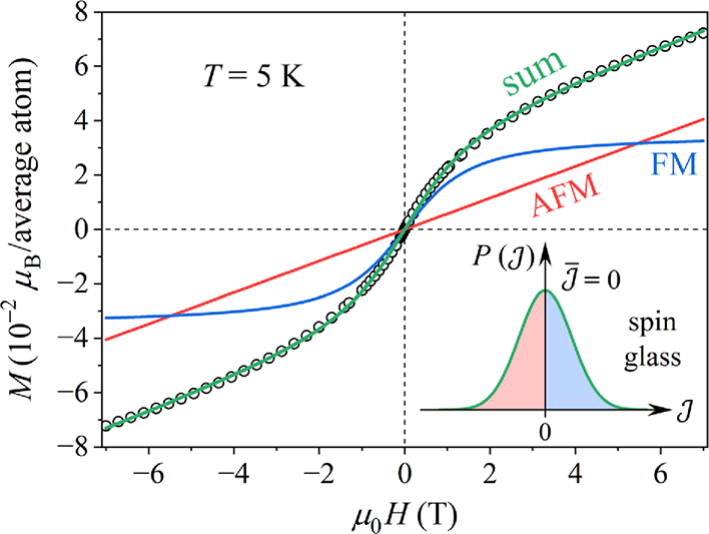
Experimental *M*(*H*) curve at *T* = 5 K (open circles) together with the theoretical fit (for details and fit parameter values, see the text). The FM and AFM contributions to the total *M* are also shown. The spin-glass distribution function of the exchange coupling constants P(J) is shown schematically in the inset.

Within this model, the *M*(*H*) curve was fitted with the simplified expression $M=M_{0}L(x)+\mu_{0}kH$ . The term $M_{0}L(x)$ accounts for the part of the P(J) distribution on the FM ( J>0 ) side of the J axis, where L(x) is the Langevin function with *x* = μμ_0_ *H*/(*k*_B_ *T*) and μ = *g*μ_B_ *S* (where *g* denotes the Landé factor and *S* is the spin). The Langevin model treats the magnetic moment as a classical vector that can assume any value (*S* → ∞), accounting for the large effective FM group spins. The Langevin function cannot reproduce the hysteresis, but just the average behavior of the *M*(*H*) curve within the hysteretic region. In the large *x* limit, the $M_{0}L(x)$ term saturates to a horizontal plateau. The term μ_0_ *kH* accounts for the part of P(J) on the AFM ( J<0 ) side, where μ_0_ *k* is the AFM susceptibility. This term is linear in *H* for any field value. The total *M*(*H*) curve therefore grows rapidly at low fields due to the Langevin function, while in the large field limit, it approaches asymptotically an inclined linear line with the slope μ_0_ *k*.

The theoretical fit of the 5 K *M*(*H*) curve is presented in Figure 9, showing good agreement with the experimental data and supporting the above SG model with mixed FM–AFM interactions. The FM and AFM contributions to the total fit are also shown separately. The fit-determined parameter values are *M*_0_ = 3.5 × 10^–2^ μ_B_/average atom, μ = 12.5 μ_B_ and *k* = 5.8 × 10^–3^ μ_B_/(average atom T).

### 3.3 Memory Effect

Below the spin-freezing temperature *T*_f_, ergodicity of the magnetically frustrated SG system is broken, because thermal spin fluctuations become too slow to drive the spin system into thermal equilibrium within the observation time window of the experimental technique. The nonergodic spin system is out of equilibrium and the observable physical quantities become time-dependent instead of being time-independent thermodynamic quantities. On the experimental time scale, the spin system slowly approaches thermal equilibrium, but cannot reach it because it does not have enough time to explore the entire phase space.

A spectacular manifestation of the out-of-equilibrium dynamics of a nonergodic spin system is the memory effect (ME).^35,40–53^ In the ME experiment, the spin system is continuously cooled in zero magnetic field from the paramagnetic (ergodic) phase through *T*_f_ into the nonergodic SG phase. At a certain temperature *T*_1_ < *T*_f_, isothermal aging of the spin system is performed by stopping the cooling for a waiting (aging) time *t*_w_ that ranges from minutes to hours. After *t*_w_, the cooling is resumed and eventually one or more additional aging stops are performed at lower temperatures *T*_i_ < *T*_1_. At the lowest temperature, a tiny magnetic field of the order 0.1 mT is applied, the cooling is reversed to heating and the zfc magnetization *M*_zfc_ is measured in a continuous heating run. The ME is manifested in the *M*_zfc_, which shows a diminution (a dip) at all stop temperatures *T*_i_ relative to the *M*_zfc_ for the no-stop case (no aging at any temperature), and the diminution is deeper for longer *t*_w_. The out-of-equilibrium spin system remembers all aging stops during cooling and also the duration *t*_w_ of each stop. By heating above *T*_f_ into the ergodic phase, all the memorized information is erased and the spin system is “rejuvenated”, i.e., it is the same as before aging.

The ME in the Fe_1.5_Ni_1.5_Ga_4_ intermetallic compound was investigated by performing the aging stop at *T*_1_= 4 K and employing a set of seven approximately logarithmically spaced aging times *t*_w_ between 3 min and 8 h, where each *t*_w_ was used in a separate experiment. A no-stop (*t*_w_ = 0) reference run was also conducted. After *t*_w_, the cooling has resumed to the lowest temperature of 1.8 K, where the magnetic field μ_0_ *H* = 0.5 mT was switched on and *M*_zfc_ was recorded in a heating run. The *M*_zfc_ s of all experiments with different *t*_w_ s are shown superimposed in Figure 10a, where it is observed that the aged *M*_zfc_ curves exhibit a dip at the aging temperature *T*_1_ = 4 K relative to the reference one.

**Figure 10:**
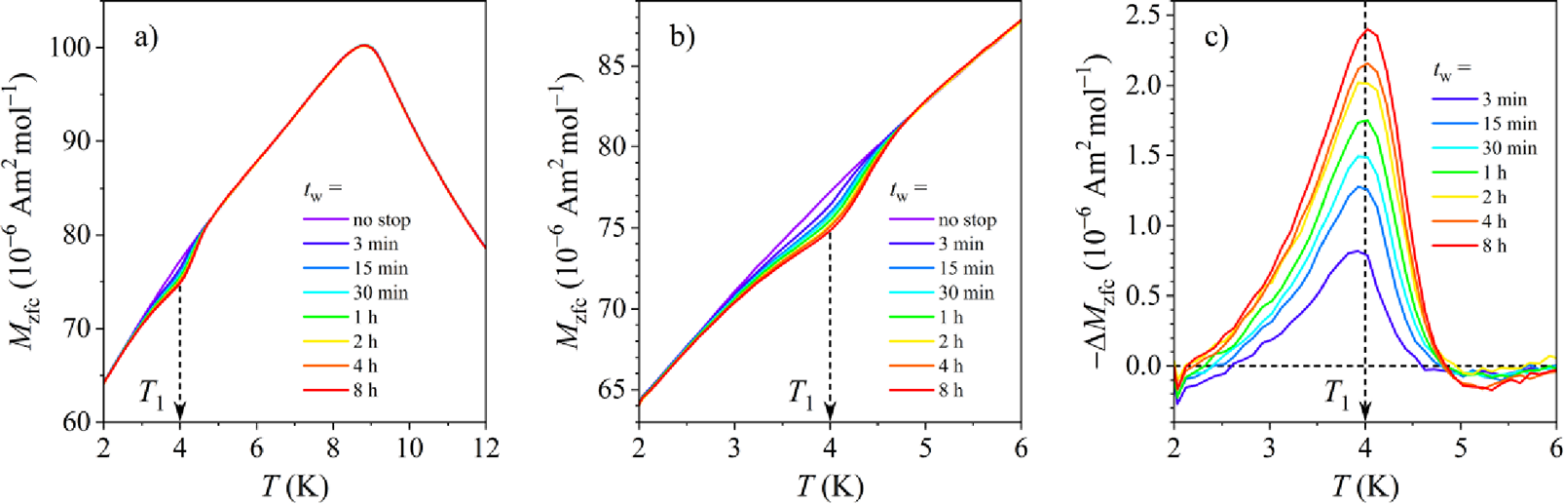
(a) *M*_zfc_ curves for the aging stop at *T*_1_ = 4 K using different aging times *t*_w_ between 3 min and 8 h. (b) Expanded *M*_zfc_ curves in the region of *T*_1_. (c) Difference Δ*M*_zfc_ between the reference (unaged) curve and the aged curves (for definition, see the text).

The expanded *M*_zfc_ curves around 4 K are shown in Figure 10b, where the monotonous increase of the dip with increasing *t*_w_ can be seen in more detail. In Figure 10c, the difference between the reference *M*_zfc_(*t*_w_ = 0) and the aged *M*_zfc_ s, Δ*M*_zfc_ = *M*_zfc_(*t*_w_) – *M*_zfc_(*t*_w_ = 0), is shown, where it is evident that Δ*M*_zfc_ appears in the form of a resonant curve peaked at the aging temperature 4 K and its magnitude increases with *t*_w_.

In the second set of experiments, the temperature dependence of ME was investigated by conducting the above-described single-stop experiment at a set of aging temperatures *T*_1_ between 9 and 3 K with the interval Δ*T*_1_ = 1 K, and in addition at 2.5 K. The aging time *t*_w_ = 1 h was employed in each experiment. The *M*_zfc_ s for all aging temperatures, together with the reference *M*_zfc_(*t*_w_ = 0) are shown superimposed in Figure 11a. At each aging temperature *T*_1_ (except at *T*_1_ = *T*_f_ = 9 K), the *M*_zfc_(*t*_w_ = 1 h) shows a diminution with respect to the reference *M*_*zfc*_(*t*_*w*_ = 0), where the diminution is peaked at *T*_1_.

**Figure 11:**
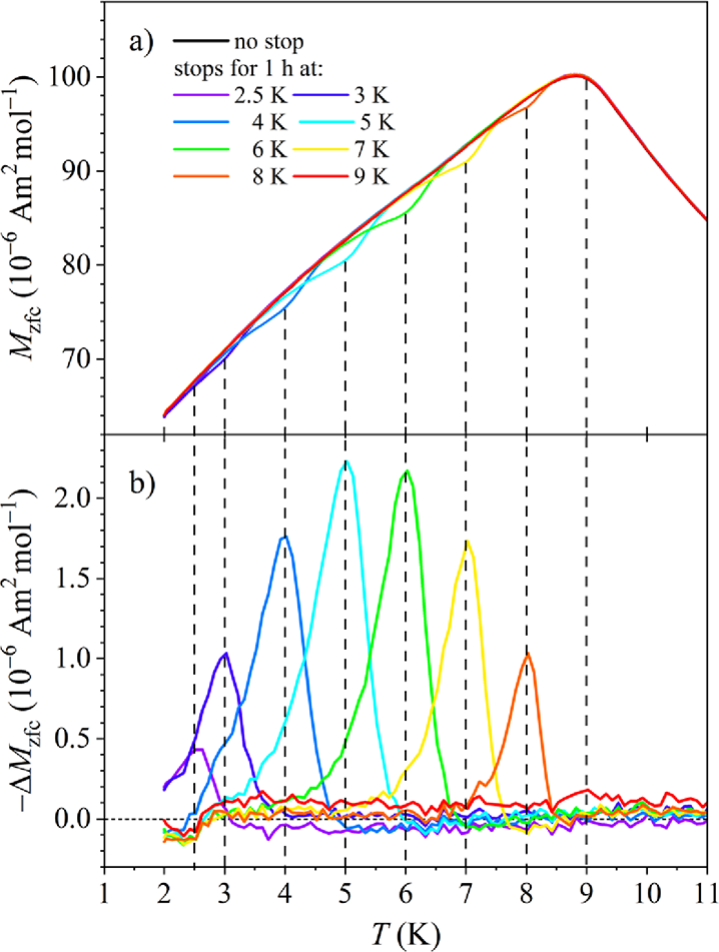
(a) *M*_zfc_ s of the single-stop experiments for the aging temperatures *T*_1_ between 9 and 3 K in steps of Δ*T*_1_ = 1 K, and in addition at 2.5 K, superimposed on the same graph. The aging time *t*_w_ = 1 h was employed in all experiments. The no-stop reference *M*_zfc_(*t*_w_ = 0) is also shown. (b) The difference Δ*M*_zfc_ between the no-stop reference magnetization and the aged magnetization for each aging temperature.

In Figure 11b, the differences Δ*M*_zfc_ for all aging temperatures are shown superimposed, demonstrating the presence of ME at any temperature below the spin freezing temperature *T*_f_, and confirming broken ergodicity of the SG state on the experimental time scale.

## 4 Discussion

As already discussed, the too similar X-ray atomic scattering factors and too close neutron coherent scattering lengths of the Fe and Ni prevent the XRD and neutron diffraction techniques to distinguish between two possible structural models of the Fe_1.5_Ni_1.5_Ga_4_ phase, the cubic *Ia*3̅*d* model with statistical distribution of the Fe and Ni atoms at their Wyckoff site and the symmetry-reduced chiral model *I*4_1_32 with chemical ordering of Fe and Ni. The observation of the low-temperature SG phase is, however, in favor of the disordered cubic *Ia*3̅*d* model via the following arguments. SGs are by definition site-disordered spin systems that conform to two basic criteria: (1) randomness (the magnetic moments of either single type or different types are positioned randomly in the crystal lattice) and (2) frustration (no moments’ configuration can satisfy all the bonds and minimize the energy at the same time).^35^ These two properties make the free-energy landscape of a SG highly structured, comprising many degenerate or nearly degenerate metastable states separated by a distribution of energy barriers. To reach the thermal-equilibrium collective spin state, the spins must explore the complete phase space by surmounting the barriers via thermally activated overbarrier jumps or by quantum tunneling through the barriers. The small thermal energy *k*_B_ *T* at low temperatures and the broad distribution of barriers between metastable states prevent the spin system to reach thermal equilibrium on the accessible experimental time scale, resulting in broken ergodicity below the spin-freezing temperature *T*_f_. The presented magnetic experiments clearly demonstrate that the Fe and Ni magnetic moments in the Fe_1.5_Ni_1.5_Ga_4_ are in a SG configuration, supporting their random distribution at the 48*g* Wyckoff site in the unit cell, which is compatible with the *Ia*3̅*d* chemically disordered structural model. Chemical ordering of the Fe and Ni atoms would result in a periodic distribution of the Fe and Ni moments on two magnetic sublattices, promoting the formation of a single periodic magnetic structure (such as FM or AFM or ferrimagnetic) without randomness. Since this has not been observed experimentally, the symmetry-reduced chiral structural model *I*4_1_32 of the Fe_1.5_Ni_1.5_Ga_4_ phase with chemically ordered Fe and Ni is unlikely.

The memory effect in frustrated spin systems is still incompletely understood, because theoretical simulations of this out-of-equilibrium phenomenon are hampered by the nonthermodynamic nature of the involved physical quantities, i.e. by the fact that the quantities depend on the observation-time window instead of being time-independent thermodynamic quantities (the latter are obtained when the observation time window is much longer than the correlation times of the thermal fluctuations). A comprehensive literature on the ME in frustrated spin systems is available,^35,40–53^ and the interested reader is referred to those articles. In the context of this study, the purpose of presenting the ME was not to add general understanding of this spectacular phenomenon, but to help distinguish between the two possible structural models of the Fe_1.5_Ni_1.5_Ga_4_ phase, where the observation of the ME favors the *Ia*3̅*d* model.

## 5 Conclusions

In this work, the Ga-rich region of the Fe–Ni–Ga ternary phase diagram was investigated, by exploring a line of compositions at a constant Ga content Fe_*x*_Ni_3–*x*_Ga_4_, with 0.5 ≤ *x* ≤ 2.5. Single-phase material was found only at the composition Fe_1.5_Ni_1.5_Ga_4_ and its immediate vicinity, representing a new phase in the Fe–Ni–Ga ternary system. The homogeneity range of the Fe_1.5_Ni_1.5_Ga_4_ phase was estimated by additionally exploring a set of compositions Fe_*x*_Ni_*y*_Ga_*z*_, where the concentrations of all three elements were varied independently around the central composition Fe_1.5_Ni_1.5_Ga_4_. The structural model of the Fe_1.5_Ni_1.5_Ga_4_ phase was constructed based on the structure of the binary Ni_3_Ga_4_ parent phase, which crystallizes in the cubic *Ia*3̅*d* space group. Despite having substantial distortions in the unit cell, we have considered that by substituting Fe for Ni, the parent cubic *Ia*3̅*d* structure is preserved in the Fe_1.5_Ni_1.5_Ga_4_, where Fe and Ni are statistically distributed at the 48*g* Wyckoff site in the unit cell. However, the possibility of a symmetry-reduced chiral structural model *I*4_1_32, driven by chemical ordering between Fe and Ni cannot be entirely ruled out on the basis of the crystallographic study performed by XRD and neutron diffraction techniques, because of insufficient X-ray scattering contrast and minimal difference in the neutron coherent scattering lengths of Fe and Ni. Magnetic properties of the Fe_1.5_Ni_1.5_Ga_4_ phase were investigated as well, finding that the material forms a spin glass phase below the spin freezing temperature *T*_f_ ≈ 9 K. Since the spin glass-type magnetic ordering of the Fe and Ni magnetic moments is compatible with their random distribution at the particular Wyckoff site in the unit cell, the magnetic study supports the disordered cubic *Ia*3̅*d* structural model of the Fe_1.5_Ni_1.5_Ga_4_ phase, without chemical ordering of the Fe and Ni.

## 6 Experimental Section

### 6.1 Single-Crystal X-ray Diffraction Data Collection and Processing

Properly shaped crystals with desired compositions were selected for the SCXRD studies. The crystals were mounted on the goniometer head using a noncrystalline adhesive. The diffraction intensities were collected by Bruker Photon II detector equipped with a monochromatic Mo Kα radiation (λ = 0.71073 Å) using Bruker D8 Quest instrument. Data was acquired with a scan width of 1° and an exposure time of 3–4 s, maintaining the sample-to-detector distance of 50 mm. Apex4 software^54^ was used for the data collection, reduction and integration. The precession image was generated by employing CrysAlisPro software.^55^ The charge flipping algorithm was used for the structure solution, implemented in Superflip^31^ embedded in the JANA2006 software.^32^ The refinement was performed using the same software.

### 6.2 EDS Analysis

Energy dispersive X-ray spectroscopy analysis of the chemical composition was performed by the scanning electron microscope ThermoFisher Verios 4G HP equipped with EDS Oxford Instruments AZtec Live, Ultim Max SDD 65 mm^2^ detection system.

### 6.3 Neutron Powder Diffraction Data Collection

Neutron powder diffraction data were collected on the Echidna high-resolution powder diffractometer with a neutron wavelength of 1.622 Å. The measurements were performed at temperatures 300 and 3 K, using ∼2 g of powder sample in a 6 mm-diameter cylindrical vanadium can.

### 6.4 Powder X-ray Diffraction Data Collection and Refinement

The phase analyses were performed using PXRD experiment. A portion of the ingot was ground into fine powder using a mortar and pestle. The samples were placed into a zero-background holder, ensuring flat upper surface for optimal data acquisition. Bruker D8 ADVANCE diffractometer with Cu Kα radiation (λ = 1.5418 Å) was used for the PXRD data collection in reflection mode for the 2*θ* range 10–80° with a scan speed of 2°/min and exposure time of 0.5 s per step. The diffraction data analyses were performed either by the Rietveld method (refining the unit cell based on the positions of the reflections, while their intensities are bound by the structure factors and a refined scale factor) or by the Le Bail method (refining the unit cell based on the positions of the reflections and adjusting the intensities individually to fit best) using the JANA2006 software.^32^

### 6.5 Magnetic Measurements

Magnetic experiments were conducted on a Quantum Design MPMS3 magnetometer, equipped with a 7 T superconducting magnet and operating at temperatures between 1.8 and 400 K. Low-field experiments were conducted using a copper AC/ULF coil of the MPMS3 magnetometer to ensure an accurate and repeatable magnetic field.

## Supplementary Material



## Notes

Reciprocal space reconstruction images, EDS elemental maps & BSE image, PXRD refinement diffractograms and associated refinement parameters (PDF)

## References

[1] Heletta L., Block T., Klenner S., Pöttgen R. (2019). Ternary transition
metal gallides with TiNiSi, ZrBeSi and MgZn_2_-type structure. Z. Naturforsch. B.

[2] Lukacheva S. M., Zakharova E. Yu., Makhaneva A. Yu., Nesterenko S. N., Kazakov S. M., Lyssenko K. A., Banaru A. M., Kuznetsov A. N. (2025). One nickel
too many: a distorted HoCoGa_5_-type structure motif and
topological heteroclusters in a ternary Ti_2‑*x*
_Ni_3_Ga_9_ intermetallic. J. Solid State Chem..

[3] Buschow K. H. J., van Engen P. G. (1981). Magnetic and magneto-optical properties
of heusler
alloys based on aluminium and gallium. J. Magn.
Magn. Mater..

[4] Xie W., Luo H., Phelan B. F., Klimczuk T., Cevallos F. A., Cava R. J. (2015). Endohedral
gallide cluster superconductors and superconductivity in ReGa_5_. Proc. Natl. Acad. Sci. U. S. A..

[5] Khalaniya R. A., Verchenko V. Yu., Zonov E. M., Stern R., Shevelkov A. V. (2024). Crystal
structure, chemical bonding, and magnetism of α-Fe_6_Ga_5_: How local interactions shape the structure and properties
of intermetallic compounds. Inorg. Chem..

[6] Gourdon O., Miller G. J. (2003). Reinvestigation
of the GaMn structure and theoretical
studies of its electronic and magnetic properties. J. Solid State Chem..

[7] Kerrai H., Saadi H., Jalal E. M., Kerouad M., Zaim A. (2024). Hysteresis
behavior and magnetocaloric effect in MnNiGa_2_ full-Heusler
alloy. Solid State Commun..

[8] Tiwari N., Pal V., Das S., Paliwal M. (2024). Proposed compositions in a Ni–Mn–Ga
system for magnetocaloric applications. J. Electron.
Mater..

[9] Sokolovskiy V. V., Sokolovskaya Yu. A., Zagrebin M. A., Buchelnikov V. D., Zayak A. T. (2019). Ternary diagrams and magnetic properties of Ni–Mn–Ga
Heusler alloys from *ab initio* and Monte Carlo studies. J. Magn. Magn. Mater..

[10] Miroshkina O. N., Sokolovskiy V. V., Zagrebin M. A., Taskaev S. V., Buchelnikov V. D. (2020). Theoretical
approach to investigation of the magnetic and magnetocaloric properties
of Heusler Ni–Mn–Ga alloys. Phys.
Solid State.

[11] Sokolovskaya Yu., Miroshkina O., Baigutlin D., Sokolovskiy V., Zagrebin M., Buchelnikov V., Zayak A. T. (2021). A ternary map of
Ni–Mn–Ga Heusler alloys from *ab initio* calculations. Metals.

[12] Saito T., Watanabe H. (2025). Magnetic and thermoelectric properties of Fe_2_CoGa Heusler compounds. Inorganics.

[13] Osswald J., Giedigkeit R., Jentoft R. E., Armbrüster M., Girgsdies F., Kovnir K., Grin Yu., Schlögl R., Ressler T. (2008). Palladium–gallium intermetallic compounds for
the selective hydrogenation of acetylene: Part I: Preparation and
structural investigation under reaction conditions. J. Catal..

[14] Osswald J., Kovnir K., Armbrüster M., Giedigkeit R., Jentoft R. E., Wild U., Grin Yu., Schlögl R. (2008). Palladium–gallium
intermetallic compounds for the selective hydrogenation of acetylene:
Part II: Surface characterization and catalytic performance. J. Catal..

[15] Armbrüster M., Kovnir K., Behrens M., Teschner D., Grin Yu., Schlögl R. (2010). Pd–Ga intermetallic compounds
as highly selective
semihydrogenation catalysts. J. Am. Chem. Soc..

[16] Ota A., Armbrüster M., Behrens M., Rosenthal D., Friedrich M., Kasatkin I., Girgsdies F., Zhang W., Wagner R., Schlögl R. (2011). Intermetallic
compound Pd_2_Ga as a selective catalyst for the semi-hydrogenation
of acetylene: From model to high performance systems. J. Phys. Chem. C.

[17] Studt F., Sharafutdinov I., Abild-Pedersen F., Elkjær C. F., Hummelshøj J. S., Dahl S., Chorkendorff I., Nørskov J. K. (2014). Discovery
of a Ni–Ga catalyst for carbon dioxide
reduction to methanol. Nat. Chem..

[18] Sharafutdinov I., Elkjær C. F., Pereira de Carvalho H.
W., Gardini D., Chiarello G. L., Damsgaard C. D., Wagner J. B., Grunwaldt J. D., Dahl S., Chorkendorff I. (2014). Intermetallic compounds of Ni and
Ga as catalysts for the synthesis of methanol. J. Catal..

[19] Wencka M., Kovač J., Dasireddy V. D. B. C., Likozar B., Jelen A., Vrtnik S., Gille P., Kim H. J., Dolinšek J. (2018). The effect
of surface oxidation on the catalytic properties of Ga_3_Ni_2_ intermetallic compound for carbon dioxide reduction. J. Anal. Sci. Technol..

[20] Singh A. K., Wang W., Panda D. P., Bagchi D., Goud D., Ray B., He J., Peter S. C. (2023). Cobalt-induced phase transformation
of Ni_3_Ga_4_ generates chiral intermetallic Co_3_Ni_3_Ga_8_. J. Am.
Chem. Soc..

[21] Ellner M., Kek S., Predel B. (1989). Ni_3_Al_4_ – A phase with
ordered vacancies isotypic to Ni_3_Ga_4_. J. Less Common Met..

[22] Ellner M., Best K. J., Jacobi H., Schubert K. (1969). Struktur von
Ni_3_Ga_4_. J. Less Common
Met..

[23] Raghavan V. (2009). Fe–Ga–Ni
(Iron–Gallium–Nickel). J. Phase
Equilib. Diffus..

[24] Zhang Y. J., Wang W. H., Zhang H. G., Liu E. K., Ma R. S., Wu G. H. (2013). Structure and magnetic properties
of Fe_2_NiZ (Z= Al, Ga,
Si and Ge) Heusler alloys. Physica B.

[25] Lu J. B., Yang H. X., Tian H. F., Zeng L. J., Ma C., Feng L., Wu G. H., Li J. Q., Jansen J. (2010). Cooperative
effect of monoclinic distortion and sinusoidal modulation in the martensitic
structure of Ni2FeGa. J. Solid State Chem..

[26] Oikawa K., Ota T., Ohmori T., Tanaka Y., Morito H., Fujita A., Kainuma R., Fukamichi K., Ishida K. (2002). Magnetic and martensitic
phase transitions in ferromagnetic Ni–Ga–Fe shape memory
alloys. Appl. Phys. Lett..

[27] Philippe M. J., Malaman B., Roques B., Courtois A., Protas J. (1975). Structures
cristallines des phases Fe3Ga4 et Cr3Ga4. Acta
Crystallogr..

[28] Kawamiya N., Adachi K. (1986). Magnetic and Mössbauer studies of metamagnetic
Fe3Ga4. J. Phys. Soc. Jpn..

[29] Dasarathy C., Hume-Rothery W. (1965). The system
iron-gallium. Proc.
R. Soc. London A.

[30] Al-Kanani H. J., Booth J. G., Ko K. Y. (2000). Magnetic phase transitions in (Fe_1–x_T_x_)_3_Ga_4_ alloys. J. Appl. Phys..

[31] Palatinus L., Chapuis G. (2007). SUPERFLIP – a computer program for the solution
of crystal structures by charge flipping in arbitrary dimensions. J. Appl. Crystallogr..

[32] Petříček V., Dušek M., Palatinus L. (2014). Crystallographic computing system
JANA2006: General features. Z. Kristallogr.—Cryst.
Mater..

[33] Hempelmann, R. Quasielastic Neutron Scattering and Solid State Diffusion (Oxford Series on Neutron Scattering in Condensed Matter); Oxford Academic: Oxford, U.K., 2000; pp 26–53 (Ch. 3).

[34] Yannello V. J., Fredrickson D. C. (2015). Generality of the 18-*n* rule: Intermetallic
structural chemistry explained through isolobal analogies to transition
metal complexes. Inorg. Chem..

[35] Binder K., Young A. P. (1986). Spin glasses: Experimental
facts, theoretical concepts,
and open questions. Rev. Mod. Phys..

[36] Mabbs, F. E. ; Machin, D. J. Magnetism and Transition Metal Complexes; Chapman and Hall: London, U.K., 1973; p 7.

[37] Bain G. A., Berry J. F. (2008). Diamagnetic corrections
and Pascal’s constants. J. Chem. Educ..

[38] Coey, J. M. D. Magnetism and Magnetic Materials; Cambridge University Press: Cambridge, U.K., 2010; p 216.

[39] Mydosh, J. A. Spin Glasses: An Experimental Introduction; Taylor & Francis: London, U.K., 1993; p 67.

[40] Bouchaud, J.-P. ; Cugliandolo, L. F. ; Kurchan, J. ; Mézard, M. Spin Glasses and Random Fields; Young, A. P. , Ed.; World Scientific: Singapore, 1998; pp 161–224.

[41] Vincent, E. ; Hammann, J. ; Ocio, M. ; Bouchaud, J.-P. ; Cugliandolo, L. F. Complex Behaviour of Glassy Systems; Rubi, M. , Ed.; Springer-Verlag: Berlin, Germany, 1997; Vol. 492, pp 184–219.

[42] Nordblad, P. ; Svedlindh, P. Spin Glasses and Random Fields; Young, A. P. , Ed.; World Scientific: Singapore, 1998; pp 1–28.

[43] Bouchaud J.-P., Dupuis V., Hammann J., Vincent E. (2001). Separation of time
and length scales in spin glasses: Temperature as a microscope. Phys. Rev. B.

[44] Lederman M., Orbach R., Hammann J. M., Ocio M., Vincent E. (1991). Dynamics in
spin glasses. Phys. Rev. B.

[45] Chu D., Kenning G. G., Orbach R. (1995). Effect of
magnetic fields on the
relaxation of the thermoremanent magnetization in spin glasses. Philos. Mag. B.

[46] Refregier Ph., Vincent E., Hammann J., Ocio M. (1987). Ageing phenomena
in
a spin-glass: effect of temperature changes below T_g_. J. Phys. (Paris).

[47] Dolinšek J., Jagličić Z., Sato T. J., Guo J. Q., Tsai A. P. (2003). Spin freezing in
icosahedral Tb–Mg–Zn
and Tb–Mg–Cd quasicrystals. J.
Phys.: Condens. Matter.

[48] Dolinšek J., Slanovec J., Jagličić Z., Heggen M., Balanetskyy S., Feuerbacher M., Urban K. (2008). Broken ergodicity,
memory effect, and rejuvenation in Taylor-phase and decagonal Al_3_(Mn,Pd,Fe) complex intermetallics. Phys.
Rev. B.

[49] Jonason K., Vincent E., Hammann J., Bouchaud J. P., Nordblad P. (1998). Memory and
chaos effects in spin glasses. Phys. Rev. Lett..

[50] Dupuis V., Vincent E., Bouchaud J. P., Hammann J., Ito A., Katori H. A. (2001). Aging, rejuvenation,
and memory effects in Ising and
Heisenberg spin glasses. Phys. Rev. B.

[51] Dolinšek J., Feuerbacher M., Jagodič M., Jagličić Z., Heggen M., Urban K. (2009). A Thermal Memory Cell. J. Appl. Phys..

[52] Ghanta S., Das A., Jana P. P., Vrtnik S., Gačnik D., Luzar J., Jelen A., Koželj P., Wencka M., Dolinšek J. (2021). Structure and spin-glass magnetism
of the Mn_x_Ni_2_Zn_11‑x_ pseudo-binary
γ-brasses at low Mn contents. Inorg. Chem..

[53] Mondal A., Dey R., Jelen A., Koželj P., Kuila S. K., Pan R., Vrtnik S., Luzar J., Wencka M., Petrović J., Mihor P., Jagličić Z., Meden A., Jana P. P., Dolinšek J. (2024). Double helix of icosahedra structure
and spin glass magnetism of the δ-Co_2.5_Zn_17.5–*x*
_Mn_
*x*
_ (*x* = 0.4–3.5) pseudo-binary alloys. Inorg.
Chem..

[54] Bruker (2001). Bruker AXS Inc.: Madison, Wisconsin, USA.

[55] Rigaku Oxford Diffraction, 2018. CrysAlisPro Software system; Rigaku Corporation: Oxford, U.K..

