# Supplementary material for: Structure and Spin-Glass Magnetism of the Fe1.5Ni1.5Ga4 Metallic Alloy
Source: Inorg Chem. 2026 Feb 20;65(9):5049–59. doi: 10.1021/acs.inorgchem.5c05447 (PMC12977031; doi:10.1021/acs.inorgchem.5c05447)
Supplement: Supplementary file 1 [file ic5c05447_si_001.pdf]

Supporting Information

for

Structure and spin-glass magnetism of the $Fe_{1.5}Ni_{1.5}Ga_4$ metallic alloy

Krishnendu Buxi,[1] Rahul Pan,[1] Zvonko Jagličić,[2] Andreja Jelen,[3] Jože Luzar,[3]

Peter Mihor,[3] Stanislav Vrtnik,[3,4] Primož Koželj,[3,4] Julia Petrović,[3] Maxim Avdeev,[5,6]

Partha Pratim Jana,[1,§] Janez Dolinšek[3,4,*]

[1] *Department of Chemistry, Indian Institute of Technology, Kharagpur-721302, India*

[2] *Institute of Mathematics, Physics and Mechanics & University of Ljubljana, Faculty of Civil and Geodetic Engineering, Jadranska 19, SI-1000 Ljubljana, Slovenia*

[3] *Jožef Stefan Institute, Jamova 39, SI-1000 Ljubljana, Slovenia*

[4] *University of Ljubljana, Faculty of Mathematics and Physics, Jadranska 19, SI-1000 Ljubljana, Slovenia*

[5] *Australian Nuclear Science and Technology Organisation, New Illawarra Road, Lucas Heights, NSW 2234, Australia*

[6] *School of Chemistry, The University of Sydney, Sydney 2006, Australia*

[§] Corresponding author. *E-mail address*: ppj@chem.iitkgp.ac.in (P.P. Jana).

* Corresponding author. *E-mail address*: janez.dolinsek@ijs.si (J. Dolinšek).

1. *hk*0 and *hhl* planes in the reciprocal space constructed from the SCXRD data collected for the $Fe_{1.5}Ni_{1.5}Ga_4$ crystal.

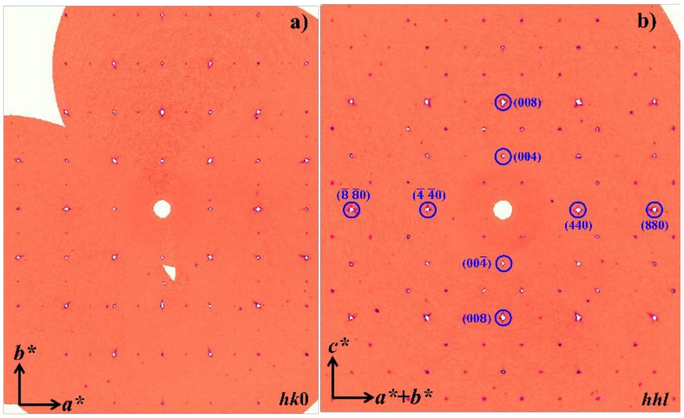

Figure S1. (a) *hk*0 and (b) *hhl* planes in the reciprocal space constructed from the SCXRD data collected for the $Fe_{1.5}Ni_{1.5}Ga_4$ crystal. The reflections corresponding to $h + k + l = 2n$ are encircled in blue.

2. EDS elemental maps of the $Fe_{1.5}Ni_{1.5}Ga_4$ material.

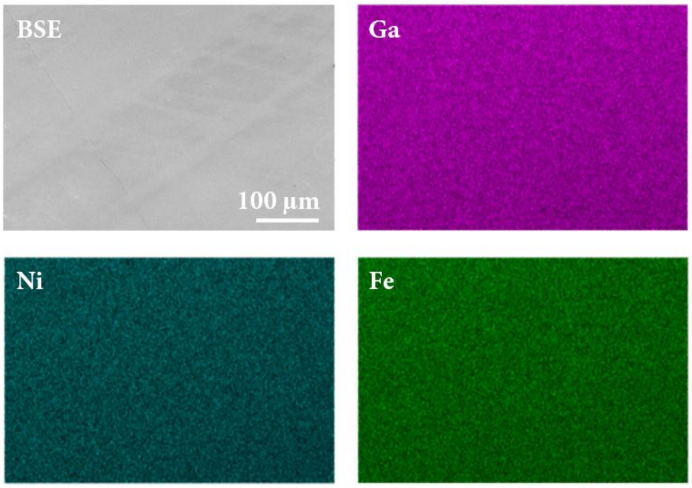

Figure S2. EDS elemental maps of the $Fe_{1.5}Ni_{1.5}Ga_4$ material. The SEM backscattered-electron (BSE) image of the same area is shown in the upper left panel.

3. Refinement diffractograms (either Rietveld or Le Bail) and refinement details of PXRD data of ten $Fe_xNi_{3-x}Ga_4$ compositions along a constant Ga line (57.14 at.% of Ga).

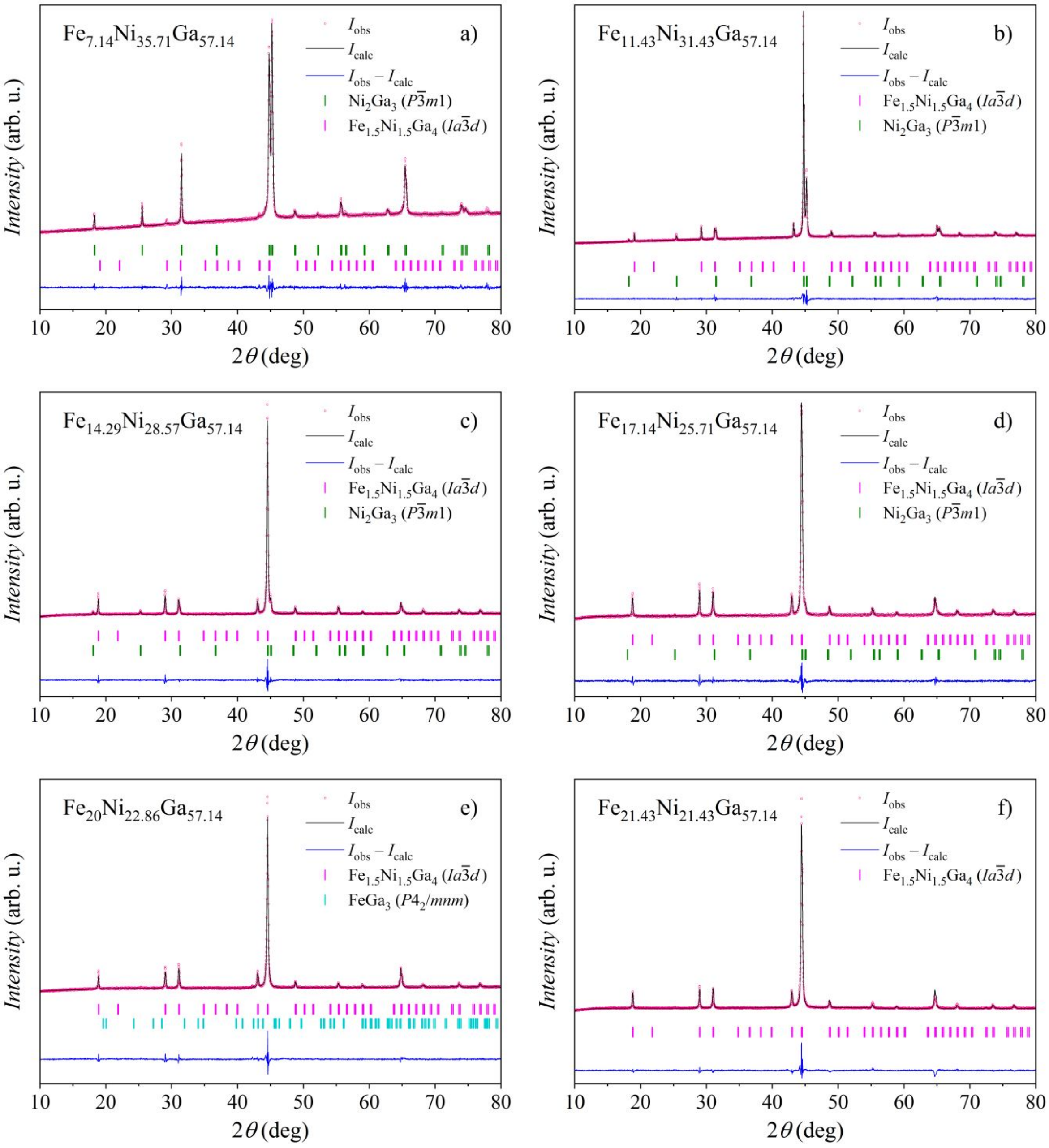

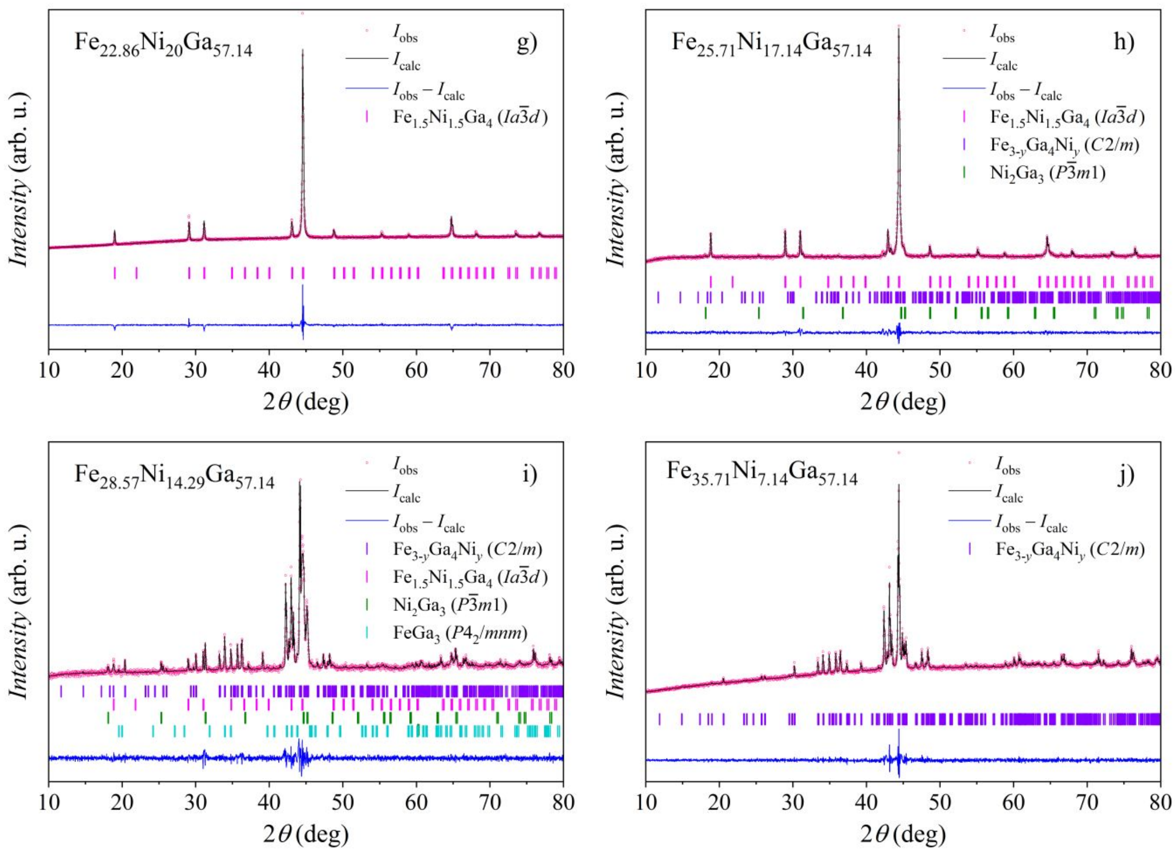

Figure S3. Refinement diffractograms (either Rietveld or Le Bail) of PXRD data of ten $Fe_xNi_{3-x}Ga_4$ compositions along a constant Ga line (57.14 at.% of Ga). The loading compositions (in at.%) with nominal Fe contents x = 0.5, 0.8, 1.0, 1.2, 1.4, 1.5, 1.6, 1.8, 2.0 and 2.5 are written in the top left corner of each panel. Bragg positions of the phases present in each composition are shown by tick marks (in different color for each phase) and are ordered according to their presence (the top trace is the major phase). The refinement parameters for each composition are given in Table S1.

Table S1. Refinement details of the $Fe_xNi_{3-x}Ga_4$ samples. For each composition, the major phase is denoted as P1, while other phases are denoted as P2, P3, and P4 (with decreasing presence).

**Composition (at.%)**			**Phases**	**Refinement method**	**Refinement parameters**
Fe	Ni	Ga			
7.14	35.71	57.14	P1: Ni_2Ga_3 ($P\bar{3}m1$)<br>P2: $Fe_{1.5}Ni_{1.5}Ga_4$ ($Ia\bar{3}d$)	Le Bail	GOF =1.29, R_p = 0.0293, wR_p = 0.0385
11.43	31.43	57.14	P1: $Fe_{1.5}Ni_{1.5}Ga_4$ ($Ia\bar{3}d$)<br>P2: Ni_2Ga_3 ($P\bar{3}m1$)	Le Bail	GOF =1.45, R_p = 0.0305, wR_p = 0.0417
14.29	28.57	57.14	P1: $Fe_{1.5}Ni_{1.5}Ga_4$ ($Ia\bar{3}d$)<br>P2: Ni_2Ga_3 ($P\bar{3}m1$)	Le Bail	GOF =1.53, R_p = 0.0221, wR_p = 0.0307
17.14	25.71	57.14	P1: $Fe_{1.5}Ni_{1.5}Ga_4$ ($Ia\bar{3}d$)<br>P2: Ni_2Ga_3 ($P\bar{3}m1$)	Le Bail	GOF =1.43, R_p = 0.0211, wR_p = 0.0288
20.00	22.86	57.14	P1: $Fe_{1.5}Ni_{1.5}Ga_4$ ($Ia\bar{3}d$)<br>P2: $FeGa_3$ ($P4_2/mnm$)	Le Bail	GOF =1.53, R_p = 0.0242, wR_p = 0.0335
21.43	21.43	57.14	P1: $Fe_{1.5}Ni_{1.5}Ga_4$ ($Ia\bar{3}d$)	Rietveld	GOF =1.88, R_p = 0.0189, wR_p = 0.0292
22.86	20.00	57.14	P1: $Fe_{1.5}Ni_{1.5}Ga_4$ ($Ia\bar{3}d$)	Rietveld	GOF =1.68, R_p = 0.0302, wR_p = 0.0443
25.71	17.14	57.14	P1: $Fe_{1.5}Ni_{1.5}Ga_4$ ($Ia\bar{3}d$)<br>P2: $Fe_{3-y}Ga_4Ni_y$ ($C2/m$)<br>P3: Ni_2Ga_3 ($P\bar{3}m1$)	Le Bail	GOF =1.22, R_p = 0.0194, wR_p = 0.0252
28.57	14.29	57.14	P1: $Fe_{3-y}Ga_4Ni_y$ ($C2/m$)<br>P2: $Fe_{1.5}Ni_{1.5}Ga_4$ ($Ia\bar{3}d$)<br>P3: Ni_2Ga_3 ($P\bar{3}m1$)<br>P4: $FeGa_3$ ($P4_2/mnm$)	Le Bail	GOF =1.39, R_p = 0.0213, wR_p = 0.0291
35.71	7.14	57.14	P1: $Fe_{3-y}Ga_4Ni_y$ ($C2/m$)	Le Bail	GOF =1.27, R_p = 0.0239, wR_p = 0.0319

4. Refinement diffractograms (either Rietveld or Le Bail) and refinement details of PXRD data of fourteen $Fe_xNi_yGa_z$ compositions ($1.3 \le x \le 1.74$, $1.26 \le y \le 1.7$ and $3.8 \le z \le 4.2$).

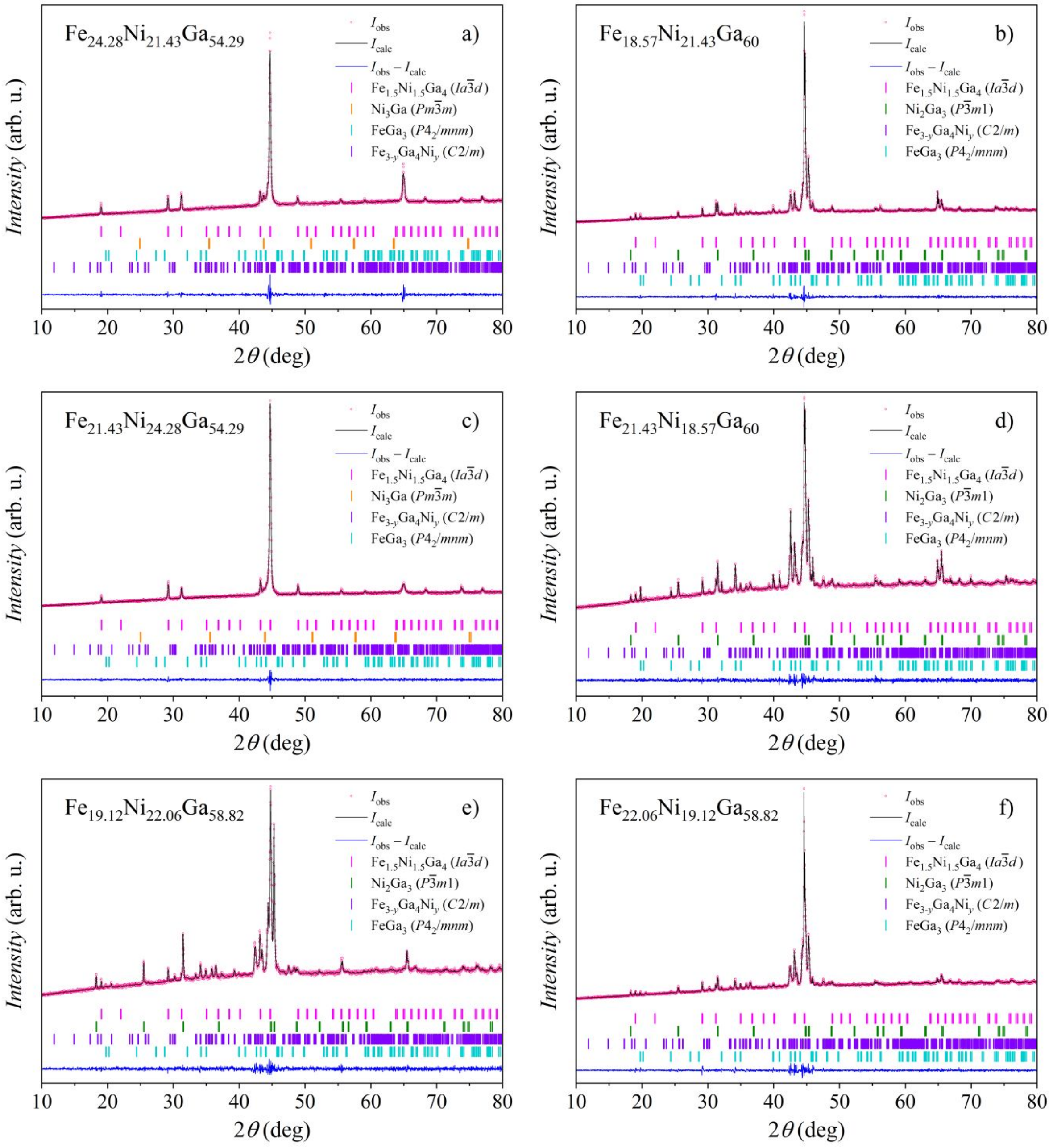

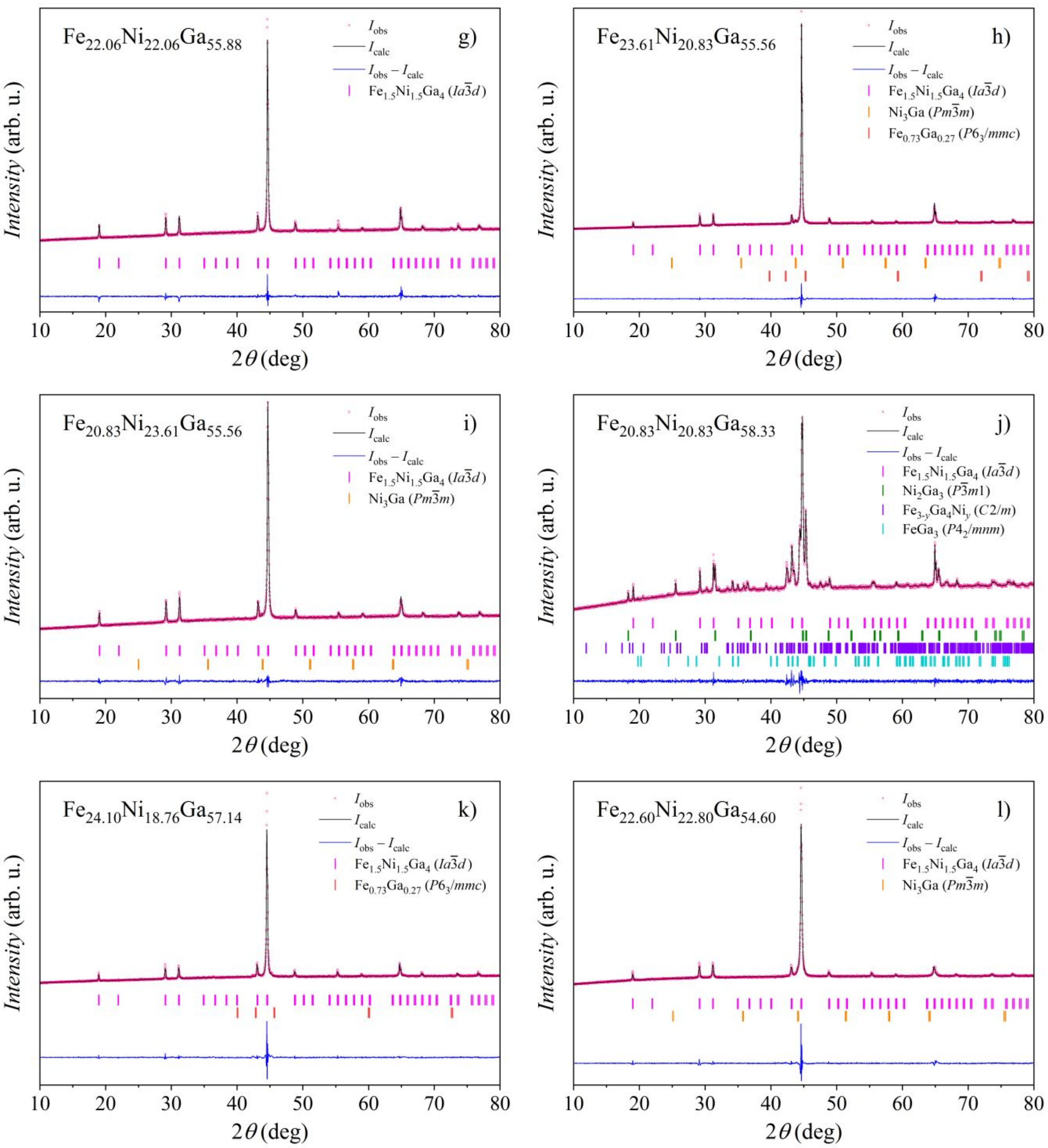

Fe22.06Ni22.06Ga55.88
g)
Iobs
Icalc
Iobs − Icalc
Fe1.5Ni1.5Ga4 (Ia3̄d)
Fe23.61Ni20.83Ga55.56
h)
Ni3Ga (Pm3̄m)
Fe0.73Ga0.27 (P63/mmc)
Fe20.83Ni23.61Ga55.56
i)
Fe20.83Ni20.83Ga58.33
j)
Ni2Ga3 (P3̄m1)
Fe3-yGa4Niy (C2/m)
FeGa3 (P42/mnm)
Fe24.10Ni18.76Ga57.14
k)
Fe22.60Ni22.80Ga54.60
l)
Intensity (arb. u.)
2θ (deg)
10
20
30
40
50
60
70
80

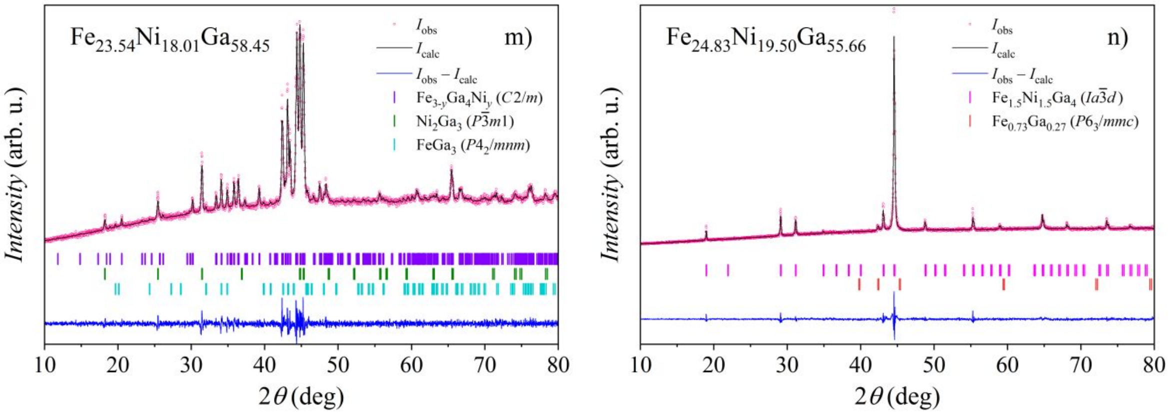

Figure S4. Refinement diffractograms (either Rietveld or Le Bail) of PXRD data of fourteen $Fe_xNi_yGa_z$ compositions ($1.3 \leq x \leq 1.74$, $1.26 \leq y \leq 1.7$ and $3.8 \leq z \leq 4.2$). The loading compositions (in at.%) are written in the top left corner of each panel. Bragg positions of the phases present in each composition are shown by tick marks (in different color for each phase) and are ordered according to their presence (the top trace is the major phase). The refinement parameters for each composition are given in Table S2.

Table S2. Refinement details of the $Fe_xNi_yGa_z$ samples, where the Ga composition was tuned along with the Fe and Ni. For each composition, the major phase is denoted as P1, while other phases are denoted as P2, P3, and P4 (with decreasing presence).

**Composition (at.%)**			**Phases**	**Refinement method**	**Refinement parameters**
Fe	Ni	Ga			
24.28	21.43	54.29	P1: $Fe_{1.5}Ni_{1.5}Ga_4$ ($Ia\overline{3}d$)<br>P2: Ni_3Ga ($Pm\overline{3}m$)<br>P3: $FeGa_3$ ($P4_2/mnm$)<br>P4: $Fe_{3-y}Ga_4Ni_y$ ($C2/m$)	Le Bail	GOF =1.28, R_p = 0.0280, wR_p = 0.0378

18.57	21.43	60.00	P1: $Fe_{1.5}Ni_{1.5}Ga_4$ ($Ia\bar{3}d$)<br>P2: Ni_2Ga_3 ($P\bar{3}m1$)<br>P3: $Fe_{3\text{-}y}Ga_4Ni_y$ ($C2/m$)<br>P4: $FeGa_3$ ($P4_2/mnm$)	Le Bail	GOF =1.32, R_p = 0.0272, wR_p = 0.0366
21.43	24.28	54.29	P1: $Fe_{1.5}Ni_{1.5}Ga_4$ ($Ia\bar{3}d$)<br>P2: Ni_3Ga ($Pm\bar{3}m$)<br>P3: $Fe_{3\text{-}y}Ga_4Ni_y$ ($C2/m$)<br>P4: $FeGa_3$ ($P4_2/mnm$)	Le Bail	GOF =1.15, R_p = 0.0263, wR_p = 0.0340
21.43	18.57	60.00	P1: $Fe_{1.5}Ni_{1.5}Ga_4$ ($Ia\bar{3}d$)<br>P2: Ni_2Ga_3 ($P\bar{3}m1$)<br>P3: $Fe_{3\text{-}y}Ga_4Ni_y$ ($C2/m$)<br>P4: $FeGa_3$ ($P4_2/mnm$)	Le Bail	GOF =1.16, R_p = 0.0242, wR_p = 0.0317
19.12	22.06	58.82	P1: $Fe_{1.5}Ni_{1.5}Ga_4$ ($Ia\bar{3}d$)<br>P2: Ni_2Ga_3 ($P\bar{3}m1$)<br>P3: $Fe_{3\text{-}y}Ga_4Ni_y$ ($C2/m$)<br>P4: $FeGa_3$ ($P4_2/mnm$)	Le Bail	GOF =1.15, R_p = 0.0250, wR_p = 0.0320
22.06	19.12	58.82	P1: $Fe_{1.5}Ni_{1.5}Ga_4$ ($Ia\bar{3}d$)<br>P2: Ni_2Ga_3 ($P\bar{3}m1$)<br>P3: $Fe_{3\text{-}y}Ga_4Ni_y$ ($C2/m$)<br>P4: $FeGa_3$ ($P4_2/mnm$)	Le Bail	GOF =1.46, R_p = 0.0271, wR_p = 0.0376
22.06	22.06	55.88	P1: $Fe_{1.5}Ni_{1.5}Ga_4$ ($Ia\bar{3}d$)	Rietveld	GOF =1.57, R_p = 0.0300, wR_p = 0.0441
23.61	20.83	55.56	P1: $Fe_{1.5}Ni_{1.5}Ga_4$ ($Ia\bar{3}d$)<br>P2: Ni_3Ga ($Pm\bar{3}m$)<br>P3: $Fe_{0.73}Ga_{0.27}$ ($P6_3/mmc$)	Le Bail	GOF =1.41, R_p = 0.0260, wR_p = 0.0354
20.83	23.61	55.56	P1: $Fe_{1.5}Ni_{1.5}Ga_4$ ($Ia\bar{3}d$)<br>P2: Ni_3Ga ($Pm\bar{3}m$)	Le Bail	GOF =1.29, R_p = 0.0264, wR_p = 0.0356

20.83	20.83	58.33	P1: $Fe_{1.5}Ni_{1.5}Ga_4$ ($Ia\bar{3}d$)<br>P2: Ni_2Ga_3 ($P\bar{3}m1$)<br>P3: $Fe_{3-y}Ga_4Ni_y$ ($C2/m$)<br>P4: $FeGa_3$ ($P4_2/mnm$)	Le Bail	GOF =1.26, R_p = 0.0218, wR_p = 0.0293
24.10	18.76	57.14	P1: $Fe_{1.5}Ni_{1.5}Ga_4$ ($Ia\bar{3}d$)<br>P2: $Fe_{0.73}Ga_{0.27}$ ($P6_3/mmc$)	Le Bail	GOF =1.81, R_p = 0.0290, wR_p = 0.0421
22.60	22.80	54.60	P1: $Fe_{1.5}Ni_{1.5}Ga_4$ ($Ia\bar{3}d$)<br>P2: Ni_3Ga ($Pm\bar{3}m$)	Le Bail	GOF =1.73, R_p = 0.0247, wR_p = 0.0367
23.54	18.01	58.45	P1: $Fe_{3-y}Ga_4Ni_y$ ($C2/m$)<br>P2: Ni_2Ga_3 ($P\bar{3}m1$)<br>P3: $FeGa_3$ ($P4_2/mnm$)	Le Bail	GOF =1.34, R_p = 0.0247, wR_p = 0.0328
24.83	19.50	55.66	P1: $Fe_{1.5}Ni_{1.5}Ga_4$ ($Ia\bar{3}d$)<br>P2: $Fe_{0.73}Ga_{0.27}$ ($P6_3/mmc$)	Le Bail	GOF =1.45, R_p = 0.0297, wR_p = 0.0403
